# Imaging of translocator protein upregulation is selective for pro‐inflammatory polarized astrocytes and microglia

**DOI:** 10.1002/glia.23716

**Published:** 2019-09-03

**Authors:** Maria Pannell, Vasiliki Economopoulos, Thomas C. Wilson, Veerle Kersemans, Patrick G. Isenegger, James R. Larkin, Sean Smart, Stuart Gilchrist, Véronique Gouverneur, Nicola R. Sibson

**Affiliations:** ^1^ Department of Oncology Cancer Research UK and Medical Research Council Oxford Institute for Radiation Oncology, University of Oxford Oxford UK; ^2^ Chemistry Research Laboratory University of Oxford Oxford UK

**Keywords:** astrocytes, brain inflammation, microglia, PET, translocator protein

## Abstract

Translocator protein (TSPO) expression is increased in activated glia, and has been used as a marker of neuroinflammation in PET imaging. However, the extent to which TSPO upregulation reflects a pro‐ or anti‐inflammatory phenotype remains unclear. Our aim was to determine whether TSPO upregulation in astrocytes and microglia/macrophages is limited to a specific inflammatory phenotype. TSPO upregulation was assessed by flow cytometry in cultured astrocytes, microglia, and macrophages stimulated with lipopolysaccharide (LPS), tumor necrosis factor (TNF), or interleukin‐4 (Il‐4). Subsequently, mice were injected intracerebrally with either a TNF‐inducing adenovirus (AdTNF) or IL‐4. Glial expression of TSPO and pro‐/anti‐inflammatory markers was assessed by immunohistochemistry/fluorescence and flow cytometry. Finally, AdTNF or IL‐4 injected mice underwent PET imaging with injection of the TSPO radioligand ^18^F‐DPA‐713, followed by ex vivo autoradiography. TSPO expression was significantly increased in pro‐inflammatory microglia/macrophages and astrocytes both in vitro, and in vivo after AdTNF injection (*p* < .001 vs. control hemisphere), determined both histologically and by FACS. Both PET imaging and autoradiography revealed a significant (*p* < .001) increase in ^18^F‐DPA‐713 binding in the ipsilateral hemisphere of AdTNF‐injected mice. In contrast, no increase in either TSPO expression assessed histologically and by FACS, or ligand binding by PET/autoradiography was observed after IL‐4 injection. Taken together, these results suggest that TSPO imaging specifically reveals the pro‐inflammatory population of activated glial cells in the brain in response to inflammatory stimuli. Since the inflammatory phenotype of glial cells is critical to their role in neurological disease, these findings may enhance the utility and application of TSPO imaging.

## Main points

- TSPO expression is strongly associated with pro‐ but not anti‐inflammatory microglia, macrophages and astrocytes, in vitro and in vivo.
- PET imaging with TSPO ligand 18F‐DPA‐713 reveals the pro‐inflammatory population of glial cells.

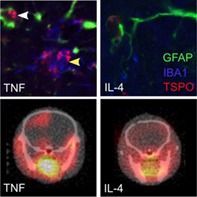

## 1 INTRODUCTION

The translocator protein (TSPO), first described as the peripheral benzodiazepine receptor (PBR), is an 18 kDa protein found predominantly on the outer mitochondrial membrane (Liu et al., 2014). TSPO is widely expressed in mammalian tissue, particularly in steroid‐producing tissues, and it has numerous functions including immunomodulation, regulation of apoptosis, cell proliferation, and steroidogenesis (Banati et al., 2014). In the CNS, however, its expression is confined to glial and ependymal cells (Banati, Myers, & Kreutzberg, 1997; Cosenza‐Nashat et al., 2009) and the healthy brain has a relatively low density of receptors (Verma & Snyder, 1989). Macrophages (Zavala, Haumont, & Lenfant, 1984), microglia (Vivash & O'Brien, 2016), and astrocytes (Lavisse et al., 2012) have all been shown to express TSPO at a low level under normal conditions, but increase this expression in neurological disease, including ischemic stroke, multiple sclerosis, Alzheimer's disease, HIV encephalitis, and cancer (Cosenza‐Nashat et al., 2009; Lavisse et al., 2012; O'Brien et al., 2014)

The increased expression of TSPO in activated glia makes it a useful tool to examine disease progression and neuroinflammation. The use of specific radiopharmaceuticals targeting TSPO in combination with positron emission tomography (PET) imaging has allowed activated glia to be observed in patients with Parkinson's disease (Ouchi et al., 2005), multiple sclerosis, glioma (Tarkkonen, Rissanen, Tuokkola, & Airas, 2016), amyloid lateral sclerosis (Turner et al., 2004), and Huntington's disease (Rupprecht et al., 2010). Although initial research utilizing PET imaging for TSPO expression focused on the expression of the receptor on activated microglia and invading peripheral macrophages, more recent studies have indicated that binding reflects upregulation of TSPO on astrocytes as well as microglia/macrophages (Lavisse et al., 2012; O'Brien et al., 2014). Interestingly, in glioma microglia were shown to have a limited contribution to the observed increase in TSPO expression, and the high TSPO receptor density was associated with the tumor tissue itself (Su et al., 2015).

It has long been established that microglia/brain macrophages can adopt distinct inflammatory phenotypes: (a) the pro‐inflammatory phenotype (historically termed M1), associated with neuroinflammation, inflammatory cytokine release (Laskin, Sunil, Gardner, & Laskin, 2011; Taylor, Jones, Kubota, & Pocock, 2005), increased phagocytosis (Fu, Shen, Xu, Luo, & Tang, 2014), and in many cases damage and destruction to healthy neurons (Fricker, Oliva‐Martín, & Brown, 2012); and (b) the anti‐inflammatory phenotype (historically termed M2), which is known as the neuroprotective phenotype and is characterized by anti‐inflammatory cytokine release (Kigerl et al., 2009), immunoregulation and tissue repair (Cairo, Recalcati, Mantovani, & Locati, 2011). It is now generally agreed, however, that the “M1/M2” distinction is far too simplistic and that microglia and macrophages undergo a spectrum of activation, which can be different depending on the type and length of stimulus, among other factors (Martinez & Gordon, 2014). Until very recently, it was unclear whether a similar pro‐ or anti‐inflammatory polarization also occurs in astrocytes. However, Liddelow and Barres (2017) have now reported evidence of a pro‐inflammatory subtype of reactive astrocytes, which they termed “A1.” The authors were able to show that activated microglia could induce the A1 phenotype through the secretion of IL‐1β, TNF, and C1q, and resulted in astrocytes that were unable to promote neuronal survival, outgrowth, synaptogenesis, and phagocytosis, as well as leading to the death of neurons and oligodendrocytes. Reactive astrocytes have also been found to overexpress TSPO after striatal injection of a lentiviris encoding the ciliary neurotrophic factor (CNTF) gene, while microglia did not show a noticeable increase (Lavisse et al., 2012)

Despite the established existence of different inflammatory phenotypes in glial cells, the extent to which TSPO upregulation reflects a pro‐ or anti‐inflammatory phenotype remains unclear. Two recent studies attempted to address this question, but with conflicting results, and in both cases only the microglial/macrophage populations were assessed (Beckers et al., 2018; Owen et al., 2017). Yet it is known that both astrocytes and microglia contribute to neurological disease, and the nature of their contribution depends critically on their inflammatory phenotype. This issue is particularly pertinent in brain cancer, where a pro‐inflammatory phenotype is known to be anti‐tumorigenic, while an anti‐inflammatory phenotype is pro‐tumorigenic. Thus, in the context of TSPO as an imaging biomarker, a clearer understanding the relationship between TSPO expression and glial or macrophage phenotype could greatly enhance the potential of this method for monitoring disease progression and response to therapy; particularly for assessing therapies aimed at reducing or modulating neuroinflammation. The aim of this study, therefore, was to determine whether TSPO receptor expression in microglia, macrophages, and astrocytes is associated with a specific inflammatory response in the brain, and with a specific astrocytic or microglial phenotype.

## 2 METHODS

### 2.1 Preparation of cultured microglia and astrocytes

Primary mixed glial cultures were prepared from the cerebral cortex and midbrain of newborn C57BL/6 mice, between postnatal Day 0 and 3 (P0–P3) as described previously (Giulian & Baker, 1985). Briefly, cortical and midbrain tissue was freed of blood vessels and meninges in Hank's balanced salt solution (HBBS), cut into 1 mm^3^ pieces, mechanically dissociated using a fire‐polished pipette and washed in Dulbecco's modified Eagle medium (DMEM) complete, as described for neonatal microglia elsewhere (Giulian & Baker, 1985). Cells were then plated in 75 cm^2^ flasks. Cultures received fresh complete DMEM medium every other day and were treated with 20 ng/ml M‐CSF after 5 days or once cells became confluent. Cultures were shaken 1 week later in a heated orbital shaker at 200 rpm for 30 min to remove microglia, which were then replated in 24 well dishes. Fresh media was added and flasks were shaken for a further 12 hr to remove oligodendrocyte precursor cells. Astrocytes were then removed using trypsin and plated in 24 well plates.

### 2.2 Culture and polarization of bone marrow‐derived macrophages

Bone marrow‐derived cells were isolated and cultured as previously described (Zhang, Goncalves, & Mosser, 2008). In summary, mice were killed by cervical dislocation and the femurs and tibias were removed, keeping the femoral head and femur intact, and cutting off the paw bones below the distal end of the tibia. Any muscle connected to the bone was carefully removed. Bones were stored in sterile HBSS on ice until all bones had been collected. After cutting through the epiphysis at both ends of the femur and tibia, sterile HBSS was slowly flushed through the bone using a 23‐G needle and 5‐ml syringe, and the content was collected in a sterile 15‐ml polypropylene tube. Cells were dissociated by briefly passing them through the syringe, followed by straining using a 70‐μm cell strainer. After centrifugation (4°C, 7 min at 500 g), red blood cells were lysed by adding 1 ml erythrocyte lysis buffer (QIAGEN). After 1–2 min, HBSS (20 ml) was added, the suspension was centrifuged as before, and the supernatant removed. Cells were washed in complete DMEM (20 ml) and again centrifuged. After removal of the supernatant, cells were plated in a 10‐cm dish with macrophage colony‐stimulating factor (MCSF; 10 ng/ml; PeproTech) to allow differentiation of bone marrow cells to macrophages. After 6 days, cells were seeded in either 24 well or 6 well plates at 200,000 cells per well or 600,000 cells, respectively, for cytokine release or flow cytometry experiments. For polarization of cells to an anti‐inflammatory phenotype, cells were stimulated with IL‐4 (20 ng/ml; BD Biosciences) for 24 and for the pro‐inflammatory phenotypes, cells were stimulated with LPS (100 ng/ml; InvivoGen), or TNF (20 ng/ml; PeproTech), for 24 hr. Unstimulated cells were only subjected to the media change (Gracey, Lin, Akram, Chiu, & Inman, 2013; Jablonski et al., 2015; Kigerl et al., 2009).

### 2.3 Endothelial cell line

The mouse endothelial cell line bEnd.3 was cultured in DMEM/F‐12 Ham medium, supplemented with 10% foetal bovine serum (FBS) and l‐glutamine. After reaching confluence, cells were seeded at 600,000 cells per well in a six well dish.

### 2.4 Immunocytochemical analysis of TSPO expression on astrocytes, microglia, and macrophages

Primary astrocyte cultures were passaged by washing with PBS and incubating with trypsin. Trypsin activity was blocked with glial culture medium, and then the cells were collected by sedimentation (800*g*, 5 min, 4°C). In order to confirm the presence of astrocytes in the culture, the pellet was resuspended in glial medium and plated at 100,000 per well in a 24 well plate, containing cover slips, previously sterilized with UV light (15 min each side in a CL‐1000 Ultraviolet Crosslinker, UVP). For microglia and bone marrow‐derived macrophages, cells were plated at 200,000 per well in 24 well plates, following removal of microglia from mixed glial cultures by shaking or through removal of cultured macrophages using a cell scraper. After 48 hr growth, cells were removed from the incubator for fixation. All subsequent steps were performed at room temperature. Wells were washed with PBS and fixed with 4% w/v paraformaldehyde (PFA) in phosphate buffer for 10 min. Cells were permeabilized with PBS supplemented with 0.025% v/v Triton X‐100 (10 min) and then washed with PBS supplemented with PBS‐T (3 × 5 min). Nonspecific antibody binding was blocked with TNB (Tris‐NaCl blocking buffer; 0.1 M Tris‐HCl pH 7.5, 0.15 M NaCl, 0.5% w/v Blocking Reagent, Perkin Elmer, UK) for 30 min, prior to incubation overnight at 4°C with rat anti‐mouse GFAP (1:100, abcam) for astrocyte cultures, or rat anti‐mouse F4/80 for microglia and macrophage cultures, and rabbit anti‐PBR (1:100, abcam) for all cultures. Primary antibody binding was detected using a goat anti‐rabbit biotinylated antibody (1:100, 30 min, Vector Laboratories) followed by streptavidin‐488 (1:100, 30 min, Invitrogen) and goat anti‐rat texas red (1:100, Vector). Cover slips were mounted on Superfrost® Plus slides (Thermoscientific, UK) using DAPI mounting medium for nuclei detection (Vector Laboratories, UK). Fluorescence was visualized using a Leica SP8 confocal microscope.

### 2.5 Flow cytometry analysis of TSPO expression on astrocytes, microglia, macrophages, and endothelial cells

Astrocytes, microglia, macrophages, and endothelial cells were seeded at 600,000 per well in a six well plate for 24 hr to allow them to settle and adhere, followed by 24 and 48 hr stimulation with LPS, TNF, or IL‐4. Cells were then washed with ice‐cold FACS buffer (0.5% BSA/PBS), and removed from the plates using a cell scraper, followed by centrifugation and removal of the supernatant. All subsequent steps were performed at room temperature. Cells were fixed for 10 min in 4% PFA, followed by PBS wash, centrifugation, and permeabilization for 20 min using PBS‐T. Nonspecific antibody binding was blocked with TNB for 30 min, as above. Cells were then stained for 30 min with either anti‐GFAP‐APC, (1:10, Miltenyi) for astrocytes, anti‐F4/80‐APC antibody (1:100 eBioscience) as a general marker for microglia and macrophages or anti‐CD31 PE‐Cy7 for endothelial cells (1:200, Biolegend), and anti‐PBR‐488 for TSPO expression in all cultures (1:500, Abcam). All antibodies were prepared in FACS buffer. The staining specificity of antibodies was tested by using fluorescence minus one (FMO) controls. The percentages of positively stained cells determined over 10,000 events were analyzed using FACS Attune (ThermoFisher Scientific), and fluorescence intensity was expressed in arbitrary units on a logarithmic scale and analyzed using the FlowJo software (version 10.1r5; TreeStar, Inc.).

In order to confirm the inflammatory phenotype of microglia and astrocytes, fixed cells were incubated with anti‐MHC II‐PE‐Cy7 (1:200; eBioscience), for MHC II expression and anti‐mannose‐488 for mannose (1:100; eBioscience), as well as anti‐GFAP‐APC for astrocytes and anti‐CD11b‐eFluor450 for microglia, as above. Astrocytes were also incubated with anti‐CD109‐PE (1:100; R&D systems), as a marker of hypoxia‐induced anti‐inflammatory astrocytes, and anti‐CD14‐FITC (1:100; Abcam) and CLCF1‐FITC (1:100; Antibodies‐online) as an alternative marker of IL‐4 stimulated anti‐inflammatory astrocytes (Liddelow et al., 2017).

### 2.6 Assessment of pro‐inflammatory cytokine release by ELISA

To measure pro‐inflammatory cytokine release, cultured astrocytes were seeded at 100,000 per well in a 24 well plate and allowed to adhere for 24 hr. Cells were then stimulated with 500 μl LPS, TNF or IL‐4 as above for 24 hr. Supernatant was then removed, cells were washed twice using plain DMEM (complete) and 500 μl fresh DMEM was added. IL‐6, MCP‐1, and RANTES release was measured directly following stimulation, and 24 hr after fresh DMEM was added, using the Mouse IL‐6 ELISA Ready‐SET‐Go! Kits (eBioscience), or the Mouse CCL2/JE/MCP‐1 DuoSet ELISA and Mouse CCL5/RANTES DuoSet ELISA (R&D systems) kits according to the manufacturer's instructions.

### 2.7 Animal models

All animal experiments were approved by the UK Home Office (Animals [Scientific Procedures] Act 1986) and conducted in accordance with the University of Oxford Policy on the Use of Animals in Scientific Research and the ARRIVE guidelines (Kilkenny, Browne, Cuthill, Emerson, & Altman, 2010). Female CD57BL/6 mice were anaesthetised with 2–3% isoflurane in 70% N_2_:30% O_2_, placed in a stereotactic frame and focally microinjected via a burr hole in the skull in the left striatum (+0.5 and 1.5 mm lateral from bregma; depth 2.5 mm) with either 1 μl PBS, 100 ng IL‐4 in 1 μl PBS or 1 × 10^6^ TNF‐inducing adenovirus (AdTNF) in 0.5 μl PBS using a 50 μm‐tipped glass microcapillary (Clark Electromedical Instruments, UK). At 24 hr after IL‐4 injection, mice were transcardially perfusion‐fixed with 0.9% heparinized saline followed by 50 ml of periodate lysine PFA containing 0.1% glutaraldehyde (PLP_light_). AdTNF injected animals were kept until either Day 3 or Day 5, then perfusion‐fixed as above. In all cases, the whole brain was subsequently fixed in PLP_light_ for 4 hr, then cryprotected in 30% sucrose for 24 hr. Brain were frozen in isopentane and stored at −20°C prior to cutting and mounting for immunofluorescence staining.

### 2.8 Immunohistochemistry

Briefly, sections were quenched in methanol with 1% H_2_O_2_ (30% w/w). The tissue was subsequently permeabilized with PBS‐T, blocked using TNB and incubated with the relevant primary antibody overnight at 4°C: chicken anti‐GFAP for astrocytes (1:250, Abcam), goat anti‐Iba1 for microglia (1:250, Abcam), or rabbit anti‐PBR for TSPO (1:100, Abcam). Primary antibody binding was detected using the relevant biotinylated secondary antibody and an ABC kit (both 1:100, Vector Laboratories). Immunoreactivity was revealed using standard diaminobenzidine HCl histochemistry and sections counterstained with cresyl violet. Sections were scanned using the ScanScope CS system (Aperio). GFAP, Iba1, and TSPO staining intensity in the striatum was measured using ImageScope and quantified by normalization to the contralateral hemisphere.

### 2.9 Immunofluorescent detection of TSPO, MHC II, and Mannose

To determine astrocyte and microglial/macrophage expression of TSPO in vivo, fluorescent colocalization was used. Briefly, after antigen retrieval in citrate buffer, brain sections were permeabilized in PBS‐T and then incubated with TNB (PerkinElmer, UK) for 1 hr. Sections were incubated overnight at 4°C, followed by 1 hr at room temperature the following day with relevant primary antibodies: chicken anti‐GFAP for astrocytes (1:250, Abcam); goat anti‐Iba1 for microglia/macrophages (1:250, Abcam); rabbit anti‐PBR for TSPO (1:100, Abcam); rat anti‐MHC II (1:50, Invitrogen); or rat anti‐CD206 for mannose receptor expression (1:100, Biorad) in PBS‐T. After washing, sections were incubated with biotinylated anti‐goat IgG (1:100, Vector Laboratories) in PBS (1 hr, room temperature). Slides were washed and incubated with a streptavidin‐405 fluorophore (1:100, Invitrogen) and Alexa 647 anti‐Rabbit (1:100, Abcam) or Alexa 647 anti‐rat (1:50, Abcam) and Alexa 488 anti‐chicken (1:100, Abcam) for 1 hr. Slides were mounted using Vectashield mounting medium (Vector Laboratories).

Images were acquired using an inverted confocal microscope (LSM‐710, Carl Zeiss Microimaging, Jena, Germany) or Leica DM IRBE (Leica, Germany) attached to a camera (Hamamatsu, Japan), and analyzed using Zen (Carl Zeiss) or Simple PCI (Hamamatsu, Japan) software.

### 2.10 Isolation and flow cytometry analysis of microglia and astrocytes after intracerebral injection of cytokines

In order to confirm TSPO immunofluorescence staining in brain slices, microglia and astrocytes from the injection site were isolated and analyzed using flow cytometry. Mice were intracerebrally injected as before with IL‐4 and TNF, and perfused with saline after 24 hr for IL‐4 mice, and 3 days or 5 days for TNF mice. The brain was then placed into ice‐cold HBSS, and a 4 mm^3^ section of the cortex and striatum was dissected out from both the injected and contralateral side. The dissected tissue was then homogenized, using a manual tissue homogenizer, in ice‐cold HBSS and washed through a 70 μm cell strainer, followed by centrifugation at 500 rpm for 20 min. The cell suspension was then incubated for 15 min with Miltenyi Biotec myelin removal beads, followed by magnetic separation using one LargeScale column per sample (Miltenyi Biotec, Bergisch Gladbach, Germany). Cells were fixed using 4% PFA, followed by PBS wash. Cells were then permeabilized for 20 min at RT with PBS‐T, followed by blocking for 30 min with TNB (PerkinElmer, UK). Cells were then incubated with anti‐GFAP‐APC, for astrocytes (1:10, Miltenyi), anti‐CD11b‐eFluor450 for microglia (1:100; eBioscience), anti‐CD31‐PE‐Cy7 for endothelial cells (1:200; Biolegend) and anti‐PBR‐488 for TSPO expression (1:500, Abcam). To determine the absolute cell numbers, CountBright™ Absolute Counting Beads (ThermoFisher Scientific) were used. The absolute cell numbers and the percentage of positively stained cells were determined over 20,000 events, and the data were analyzed using the FlowJo software, as specified in Section 2.5.

In order to confirm the inflammatory phenotype in microglia and astrocytes, the cell suspensions were also incubated with anti‐MHC II‐PE‐Cy7 (1:200; eBioscience) for MHC II expression, and anti‐mannose‐488 (1:100; eBioscience) for mannose expression, as well as anti‐GFAP‐APC for astrocytes and anti‐CD11b‐eFluor450 for microglia, as above.

### 2.11 PET imaging of brain inflammation using ^18^F‐DPA‐713

Synthesis of ^18^F‐DPA‐713 was obtained via the Cu‐mediated ^18^F‐fluorodeboronation of the corresponding boronic ester precursor using methodology previously described (Preshlock et al., 2016; Taylor et al., 2017; Tredwell et al., 2014). Full materials and methods for the ^18^F‐fluorination are described in Supporting Information, Figures S1–S4 and Table S1.

PET imaging was performed using the single gantry VECTor4CT system (MIlabs, Utrecht, the Netherlands) fitted with the HE‐UHR‐RM PET/SPECT collimator (1.8 mm pinholes). Animals were anaesthetized at 3% isofluorane in air, before iv injection with 10–30 MBq of the of the TSPO ligand 18F‐DPA‐713. Mice were imaged 25 min after injection for 15 min; this static acquisition was split across 3 bed positions focused on the brain. Mice were placed supine, head first, in the imaging cradle, and maintained at ~2.5% isofluorane in air. Data were acquired in list mode (0–1,200 keV) using the MILabs acquisition software v7.39. Triple‐energy‐window based scatter and cross‐talk correction was applied for each photon peak window (photopeak: 460–562 keV with background windows set to 439–460 and 562–582 keV) and the associated calibration factor was determined to allow quantitation of PET images. All images were reconstructed with MILabs reconstruction software v3.24 on 0.8 mm isotropic 3D voxel grids using dual matrix similarity regulated ordered‐subset expectation maximization (dual matrix SROSEM; Vaissier, Beekman, & Goorden, 2016). Following reconstruction, the PET and their corresponding CT data were coregistered and resampled to equivalent 200 μm voxel sizes. CT based attenuation correction was applied. Reconstructed images were viewed and analyzed using PMOD v.3.37 (PMOD Technologies, Zurich, Switzerland). Analysis software corrected for MBq ligand dose injected. To reduce interindividual variation, all data are presented as percentage of the injected activity corrected per selected tissue volume (%IA/cm^3^). Whole body CT was performed for anatomical referencing and CT based attenuation correction. Images were acquired at 50 kV and 0.24 mA using continuous rotation (40°/s) and were reconstructed using cone‐beam filtered back projection (Feldkamp algorithm) on a 0.2 mm voxel grid. Beam hardening and ring artefacts were corrected, and the voxel numbers were converted into Hounsfield units by using a premeasured calibration factor. Regions of interest (ROIs) were drawn manually in left and right hemisphere, with the central ROIs being at the region of the brain where the skull drill hole was visible. ROIs were drawn in 10 brain sections before and after the drill hole and the ratio of intensity of left, injected versus right, un‐injected was calculated. Six animals were used per treatment for imaging and subsequent autoradiography.

### 2.12 Autoradiography

Five‐minutes following PET imaging, animals were injected with pentobarbital and transcardially perfused with heparinized saline. Brains were removed and fresh‐frozen in isopentane and kept on dry ice before transfer to a cryostat at the end of the imaging session. Fifteen micro meter thick coronal sections were cut throughout the striatum with serial sections taken for autoradiography. For autoradiography, slides were placed on Super Resolution Phosphor screens (Perkin Elmer, UK). Screens were held in autoradiography cassettes (Fisher Biotech, Pitsburg, PA) for 30 min at RT. Subsequently, the phosphor plate was developed with Perkin Elmer Cyclone Plus using the OptiQuant software (Perkin Elmer, Boston, MA). Images were analyzed using ImageJ. Images were corrected for amount of ligand injected, and time since injection. ROIs were drawn around left and right hemisphere and the ratio of intensity was calculated as for PET.

### 2.13 Cerebral blood volume analysis by magnetic resonance imaging

Cerebral blood volume (CBV) was calculated using quantitative T_2_ maps acquired using magnetic resonance imaging (MRI), both before and after i.v. injection of ultrasmall particles of iron oxide (USPIO, 30 nm diameter, synthesized in‐house, 4 mg Fe/kg). MRI was conducted at 7.0 T (Agilent Technologies Inc., Santa Clara, CA), with a 26 mm birdcage coil (Rapid Biomedical, Rimpar, Germany) and isoflurane anaesthesia. T_2_ maps were acquired by multi‐echo acquisition, TE = 12‐100 ms, TR = 3 s, 4‐shot spin‐echo echo planar imaging, matrix = 64 × 64, FOV = 32 × 32 mm. CBV maps were calculated from T_2_ maps and particle relaxivity. ROIs were drawn on each CBV map over the ipsi‐ and contralateral striata and relative CBV for each animal was calculated.

### 2.14 Statistical analysis

Statistical analyses were performed using the GraphPad Prism software (Version 5.01 for Windows; GraphPad Software Inc.). Data are expressed as mean ± *SEM*. An ANOVA was used to analyze all data and Dunnett's multiple comparison test was used as a posthoc test. Differences were considered significant at values of *p* < .05.

## 3 RESULTS

### 3.1 Increased TSPO expression on cultured glia and macrophages following pro‐ but not anti‐inflammatory, stimulation

**Figure 1:**
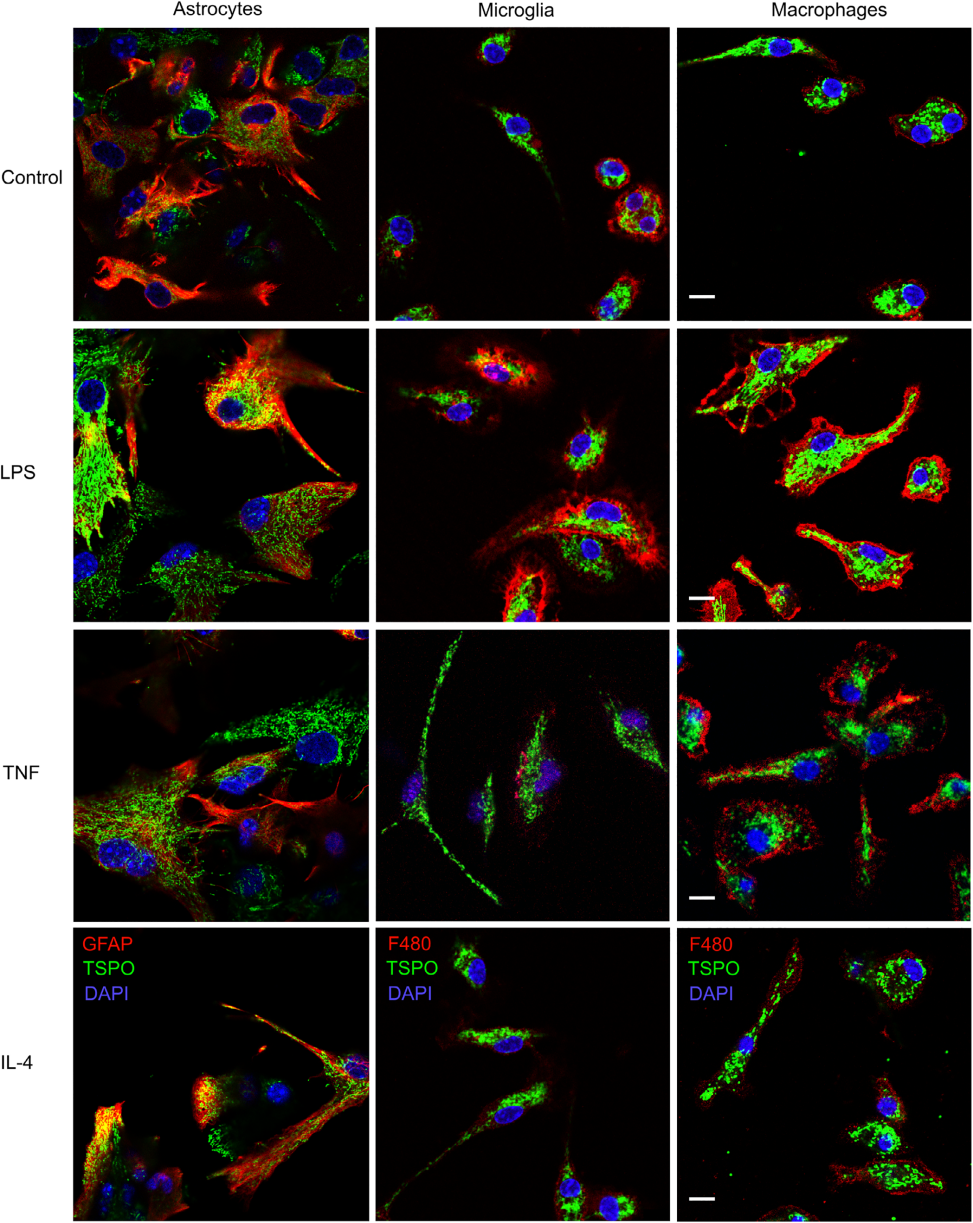
Primary cultured astrocytes, microglia, and macrophages express TSPO. Primary astrocytes and microglia cultured from C57/Bl6 mice (P0–P3), and bone marrow‐derived macrophages from adult C57/Bl6 mice (7–12 weeks). Pro‐inflammatory phenotypes were induced using LPS (100 ng/ml) or TNF (20 ng/ml), and an anti‐inflammatory phenotype using IL‐4 treatment (20 ng/ml). Astrocytes stained with anti‐GFAP‐texas red, microglia and macrophages with anti‐F4/80‐texas red. TSPO stained with anti‐PBR‐alexa 488 (green). Dapi for nuclei (blue). Scale bar 10 μm

Qualitatively, unstimulated, pro‐inflammatory (TNF) stimulated and anti‐inflammatory (IL‐4) stimulated astrocytes, microglia, and macrophages all expressed TSPO, although to differing degrees (Figure 1). Upon a pro‐inflammatory stimulus, microglia and macrophages also displayed a changed morphology, with an enlarged cell body and an increase in F4/80 expression (middle panels, LPS, and TNF treatment), while IL‐4 treated microglia and macrophages retained the morphology of untreated cells (lower panel).

**Figure 2:**
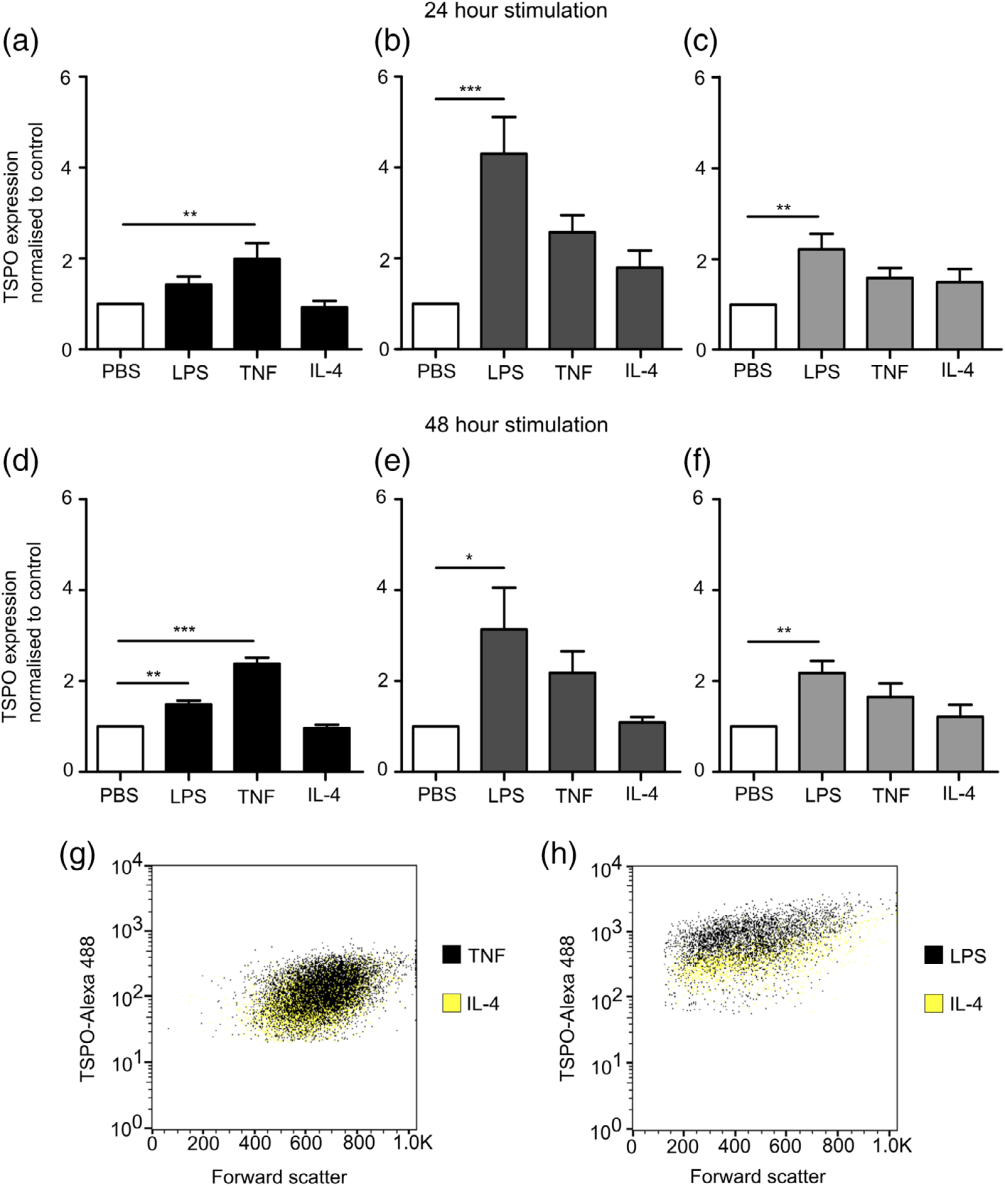
TSPO expression in glia after pro‐ and anti‐inflammatory stimulus. TSPO expression measured in primary cultured murine astrocytes (a,d), microglia (b,e), and macrophages (c,f) and measured using flow cytometry following 24 or 48 hr stimulation with either LPS, TNF, or IL‐4. Graphs show TSPO expression normalized to control (*n* > 6; ANOVA followed by Dunnett's multiple comparison test, *p < .05, **p < .005, ***p < .001). Representative dot blots showing shift in TSPO positive populations following TNF stimulation of astrocytes (g) and LPS stimulation of microglia (h), compared to IL‐4 stimulated cells

After either 24 or 48 hr of stimulation, astrocytes showed a significant increase in TSPO expression with TNF treatment (*p* < .005 and *p* < .001, respectively; Figure 2a,d), but only LPS after 48 hr stimulation (*p* < .005). In contrast, microglia and macrophages showed significant increases in TSPO expression after 24 and 48 hr of LPS stimulation (microglia *p* < .001, *p* < .05; macrophages *p* < .005, *p* < .005; Figure 2b,c,e,f)), but not TNF. IL‐4 did not cause a significant increase in TSPO expression in any of the cell types, indicating that TSPO expression is increased in cells with a pro‐inflammatory, but not anti‐inflammatory, phenotype. The endothelial cell line bEnd.3 showed low expression of TSPO in unstimulated cells, which was significantly decreased upon stimulation with LPS and IL‐4 after 24 hr stimulation (both *p* < .05; Figure S5A) and significantly decreased by LPS, TNF, and IL‐4 after 48 hr stimulation (*p* < .05, *p* < .005, *p* < .001; Figure S5B).

**Figure 3:**
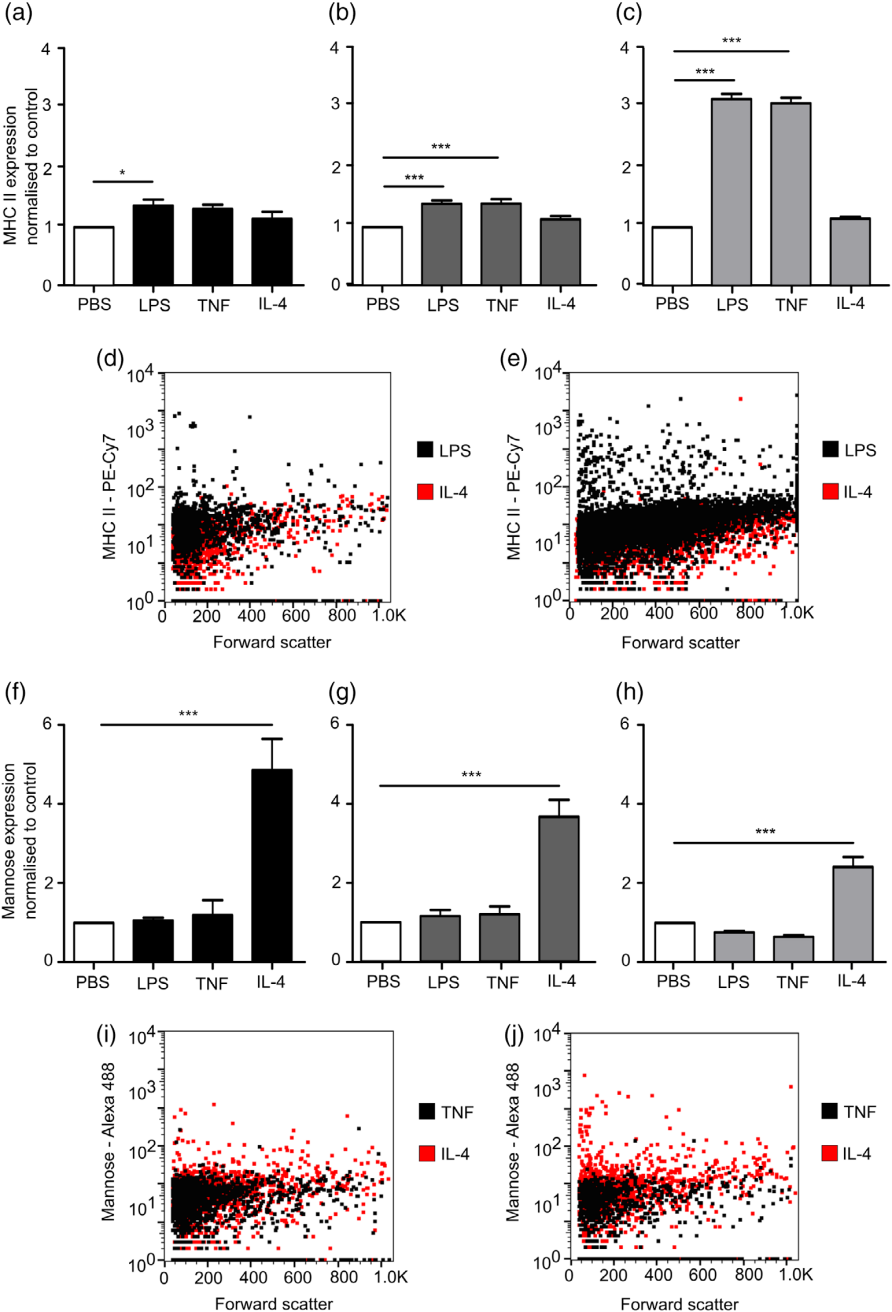
MHC II and mannose receptor expression in glia after pro‐ and anti‐inflammatory stimuli. MHC II and mannose expression measured in primary cultured murine astrocytes (a,f), microglia (b,g), and macrophages (c,h), measured using flow cytometry after 24 stimulation with either LPS, TNF, or IL‐4. Representative dot blots showing shift in MHC II positive populations following LPS stimulation of astrocytes (d) and microglia (e), compared to IL‐4 stimulated cells. Representative dot blots showing shift in mannose receptor (CD206) positive populations following IL‐4 stimulation of astrocytes (i) and microglia (j), compared to TNF‐stimulated cells. Graphs show MHC II and mannose receptor expression normalized to control (*n* > 4; ANOVA followed by Dunnett's multiple comparison test, *p < .05, **p < .005, ***p < .001)

To confirm the inflammatory phenotype of cells following LPS, TNF, or IL‐4 stimulation, we analyzed cells using flow cytometry for expression of the pro‐ and anti‐inflammatory surface markers MHC II and mannose receptor, respectively (Figure 3). Expression of CD109 as a proposed marker of hypoxia‐induced anti‐inflammatory (A2) astrocytes, and CD14 and CLCF1 as proposed markers of IL‐4‐induced anti‐inflammatory astrocytes (Liddelow et al., 2017) were also assessed. Cultured astrocytes, microglia, and macrophages all showed a significant increase in MHC II expression after LPS stimulation (*p* < .05, *p* < .001, *p* < .001; Figure 3a–c), while microglia and macrophages also showed a significant increase after TNF stimulation (*p* < .001, *p* < .001; Figure 3b,c). Mannose receptor was upregulated in all cell types following IL‐4 stimulation (*p* < .001, *p* < .001, *p* < .001; Figure 3f–h), but not LPS or TNF stimulation. CD109 expression was significantly downregulated in LPS stimulated astrocytes (*p* < .05), but unchanged in TNF and IL‐4 stimulated cells (Figure S6A). CD14 expression showed no change between treated and untreated astrocytes (Figure S6B) and CLCF1 expression was significantly reduced in IL‐4 stimulated cells (*p* < .005; Figure S6C).

**Figure 4:**
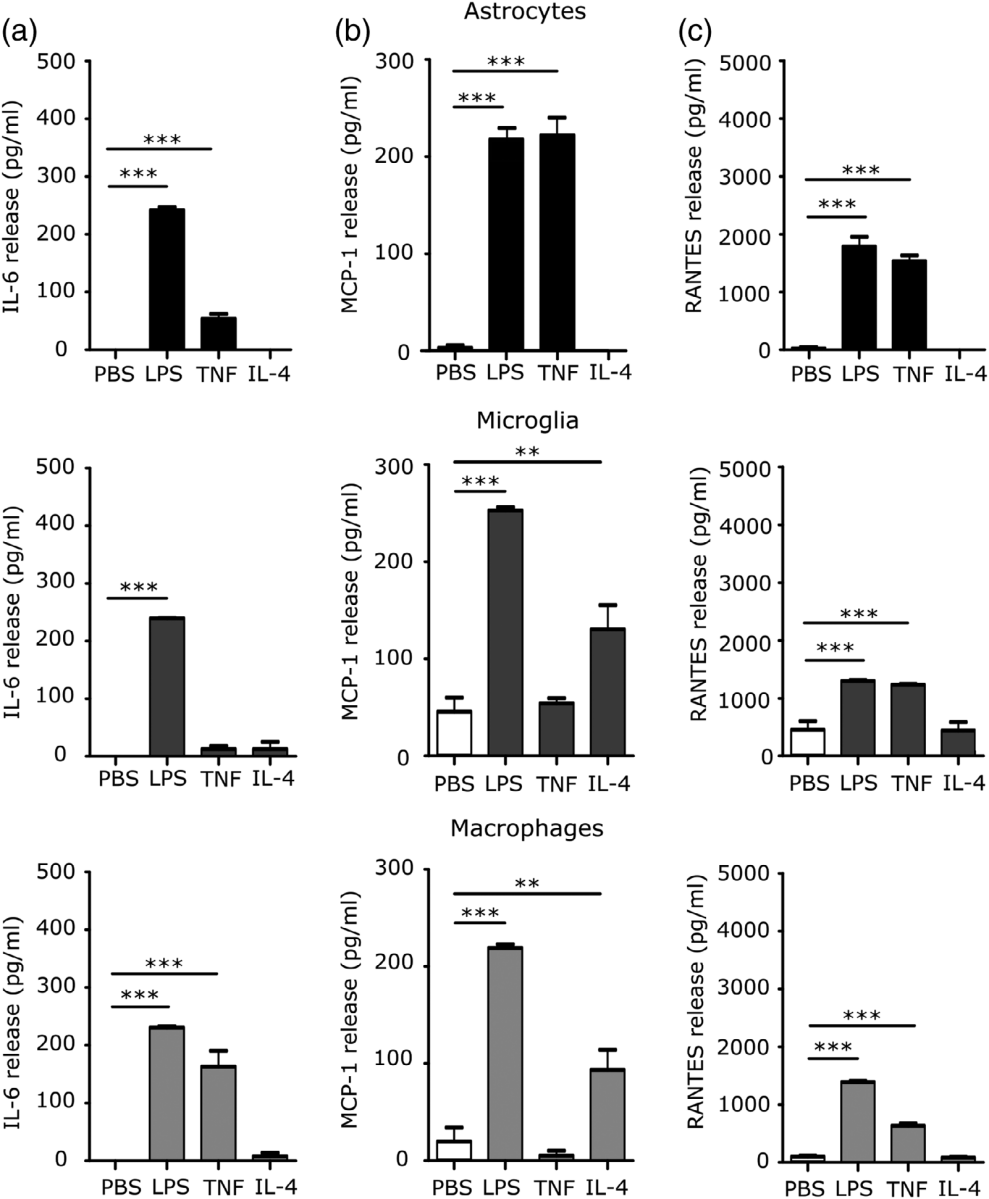
Pro‐inflammatory cytokine release in glia after pro‐ and anti‐inflammatory stimulus. (a) IL‐6, (b) MCP‐1, and (c) RANTES release from primary cultured murine astrocytes, microglia and macrophages after stimulation with LPS, TNF, or IL‐4 for 24 hr. Cytokine release into supernatant was measured directly after stimulation (*n* > 5; ANOVA followed by Dunnett's multiple comparison test, **p < .005, ***p < .001)

We also examined the release of the pro‐inflammatory cytokines IL‐6, MCP‐1, and RANTES by ELISA following stimulation with LPS, TNF, or IL‐4. Initial ANOVA confirmed significant changes in IL‐6, MCP‐1, and RANTES release across all cell groups: astrocytes IL‐6 *p* < .001, MCP‐1 *p* < .001, and RANTES *p* < .001; microglia *p* < .001 for all three; and macrophages *p* < .001 for all three. Subsequent Dunnett's posthoc tests revealed a significant increase in IL‐6 release directly after LPS stimulation in all three cell types compared to untreated cells (*p* < .001; Figure 4a)), which persisted to 24 hr (*p* < .001; Figure S7A). Following TNF stimulation, IL‐6 release was only significantly increased in macrophages and astrocytes directly after stimulation (*p* < .001; Figure 4a), compared to untreated cells. MCP‐1 release was significantly increased in LPS and TNF treated astrocytes compared to PBS treated cells directly after stimulation (*p* < .001; Figure 4b), and this was maintained to 24 hr after stimulation (Figure S7B). Both microglia and macrophages showed a significant increase in MCP‐1 release immediately after stimulation with LPS (*p* < .001; Figure 4b), but not TNF. IL‐4 treated microglia and macrophages also showed a significant upregulation of MCP‐1 release immediately after stimulation (*p* < .005; Figure 4b). LPS and TNF stimulation resulted in a significant increase in RANTES release compared to control for all cell types directly after stimulation (*p* < .001; Figure 4C), while TNF stimulation caused significant RANTES release 24 hr poststimulation in astrocytes and macrophages (*p* < .001; Figure S7C).

### 3.2 Iba1, GFAP, and TSPO staining is significantly increased following AdTNF, but not IL‐4 injection in vivo

**Figure 5:**
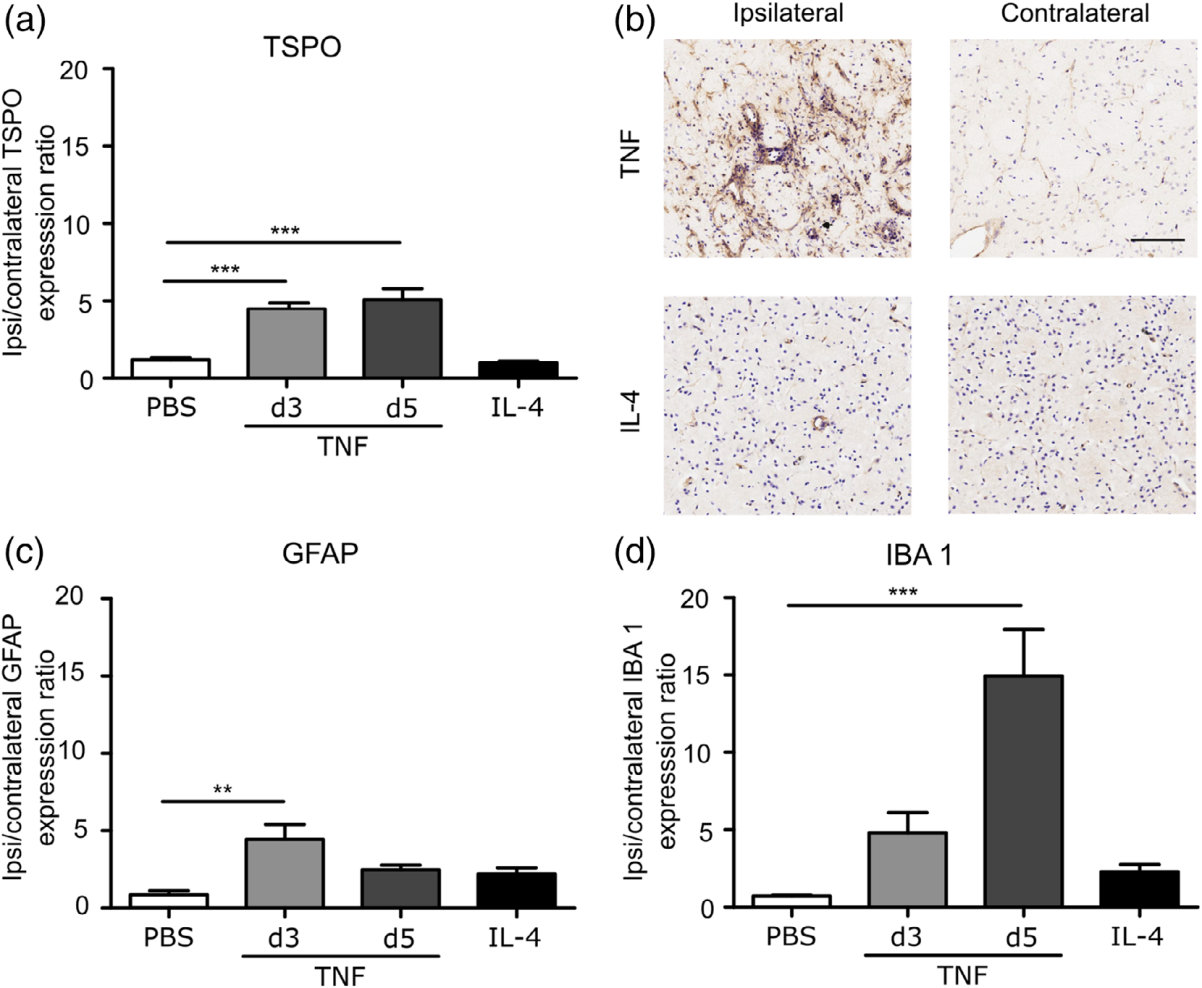
IBA1, GFAP, and TSPO staining is significantly increased after AdTNF injection. Mice were injected intracranially with either 1 × 106 AdTNF or 100 ng IL‐4 to induce a pro‐or anti‐inflammatory phenotype, respectively. (a) TSPO expression normalized to the contralateral hemisphere in AdTNF injected brains 3 or 5 days after intracerebral injection, and IL‐4 and PBS injected brains 24 hr after injection. (b) Representative immunohistochemical images showing TSPO expression (brown) in injected (ipsilateral) versus control (contralateral) hemispheres in AdTNF Day 5 versus IL‐4 injected brains. (c) GFAP expression normalized to the contralateral hemisphere in AdTNF injected brains at 3 and 5 days, and IL‐4 and PBS injected brains at 24 hr. (d) Iba1 expression normalized to the contralateral hemisphere in AdTNF injected brains at 3 and 5 days, and IL‐4 and PBS injected brains at 24 hr. (*n* = 3; ANOVA followed by Dunnett's multiple comparison test, **p < .005, ***p < .001). Scale bar = 200 μm

Immunohistochemical staining showed that TSPO expression was significantly increased in the injected hemisphere, compared to the contralateral hemisphere, at both 3 and 5 days after intrastriatal AdTNF injection (*p* < .001), but not in PBS or Il‐4 injected animals (Figure 5a,b). A significant (*p* < .005) presence of GFAP‐positive astrocytes was seen 3 days after intrastriatal AdTNF injection compared to the contralateral control striatum (Figure 5c), and a significant increase in Iba1 staining was evident 5 days after injection (*p* < .001, Figure 5d). IL‐4 injected animals showed no significant increase in either GFAP or Iba1 staining (Figure 5c,d).

### 3.3 In vivo PET imaging with ^18^F‐DPA‐713 shows increased TSPO binding after pro‐ but not anti‐inflammatory, stimulation

**Figure 6:**
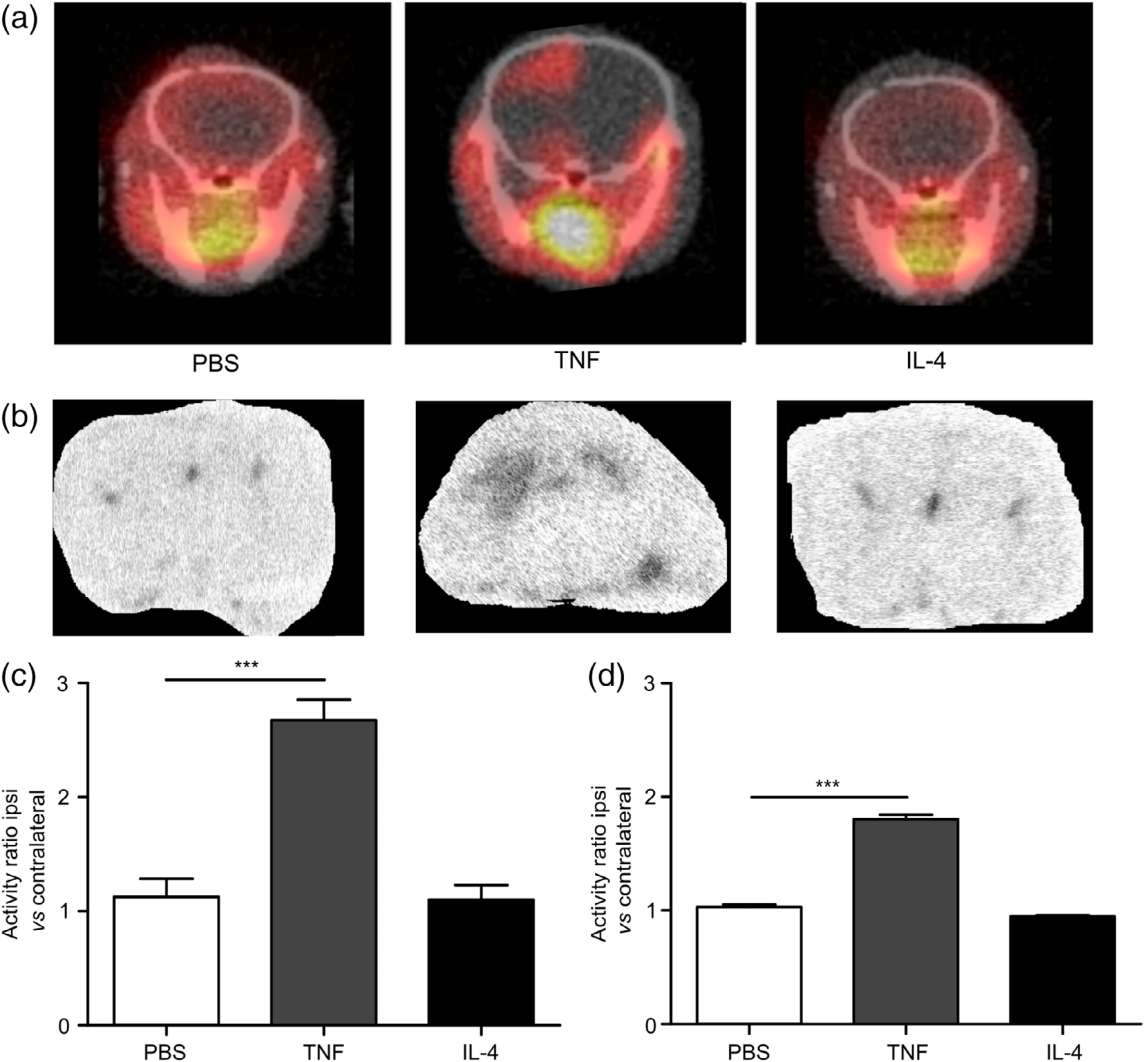
In vivo PET imaging using ^18^F TSPO ligand shows increased TSPO binding after pro‐inflammatory TNF stimulation. (a) Representative coronal PET images acquired from mice injected intracerebrally in the left striatum with either PBS, AdTNF (pro‐inflammatory), or IL‐4 (anti‐inflammatory). Imaging was performed after intravenous ^18^F‐DPA‐713 injection, to visualize TSPO expression, at either 48 hr (PBS/IL‐4) or 5 days after intracerebral injection. (b) Representative autoradiography images from the same animals, obtained after PET imaging. (c) Graph showing ratios of TSPO binding activity in the injected (ipsilateral) versus contralateral hemisphere measured from the PET data. (d) Graph showing ratios of TSPO binding activity in the injected (ipsilateral) versus contralateral hemisphere measured from the autoradiography images. (*n* = 6; ANOVA followed by Dunnett's multiple comparison test, ***p < .001)

**Figure 7:**
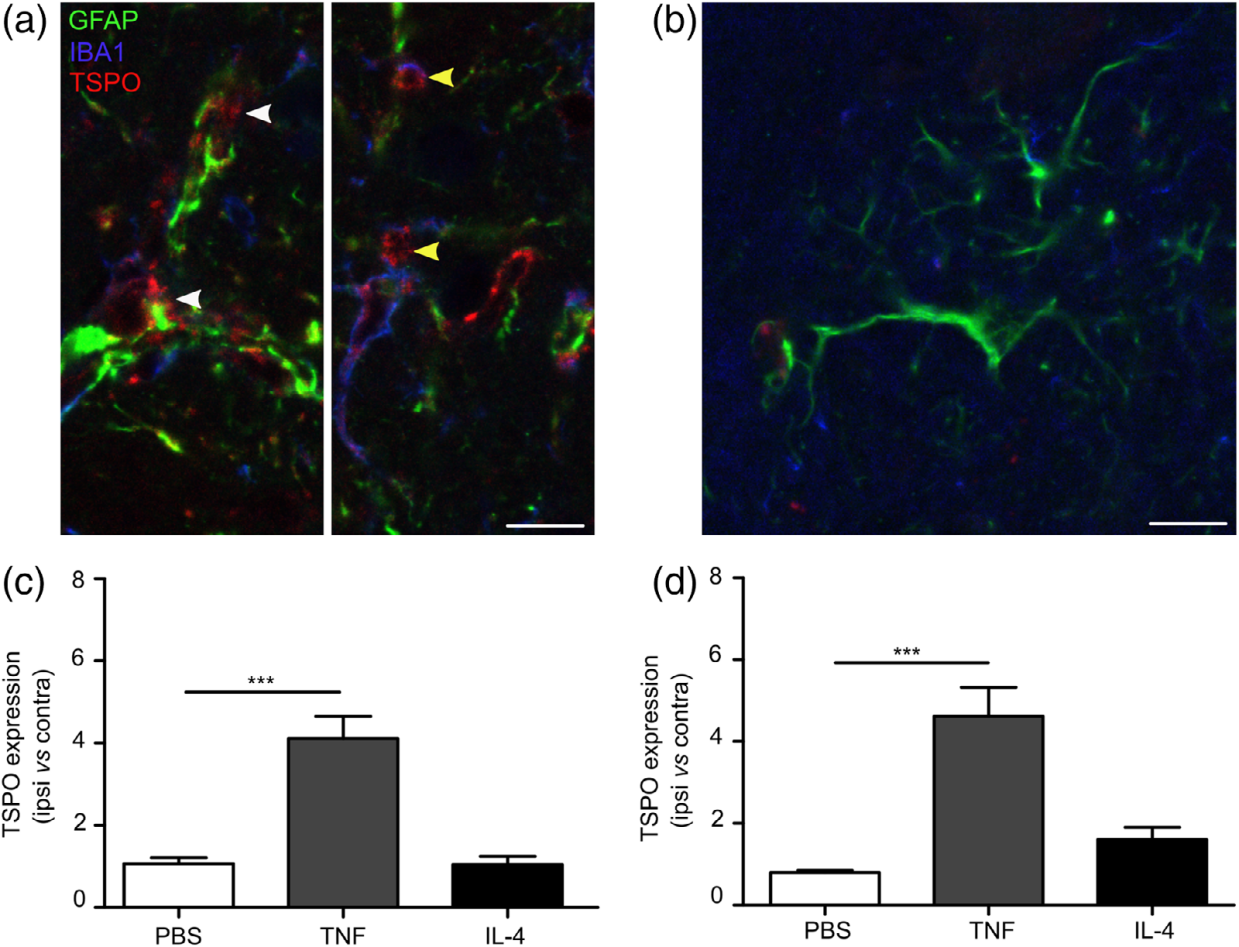
TSPO expression increases in astrocytes and microglia/macrophages in vivo after pro‐inflammatory TNF stimulation in vivo. Representative images from the injected striatum of a mouse injected intrastriatally with either (a) AdTNF or (b) IL‐4 showing Iba1 positive cells (microglia/macrophages) counterstained with alexa 405 (blue), GFAP positive cells (astrocytes) with alexa 488 (green) and TSPO with alexa 647 (red). Yellow arrow shows Iba1 (blue) colocalized with TSPO (red). White arrow shows GFAP (green) co‐localized with TSPO (red). (c) Graph showing ratios of TSPO expression in GFAP positive astrocytes in injected (ipsilateral) versus contralateral hemispheres. (d) Graph showing ratios of TSPO expression in Iba1 positive microglia/macrophages in injected (ipsilateral) versus contralateral hemispheres (*n* = 3; ANOVA followed by Dunnett's multiple comparison test, ***p < .001). Scale bar = 10 μm

**Figure 8:**
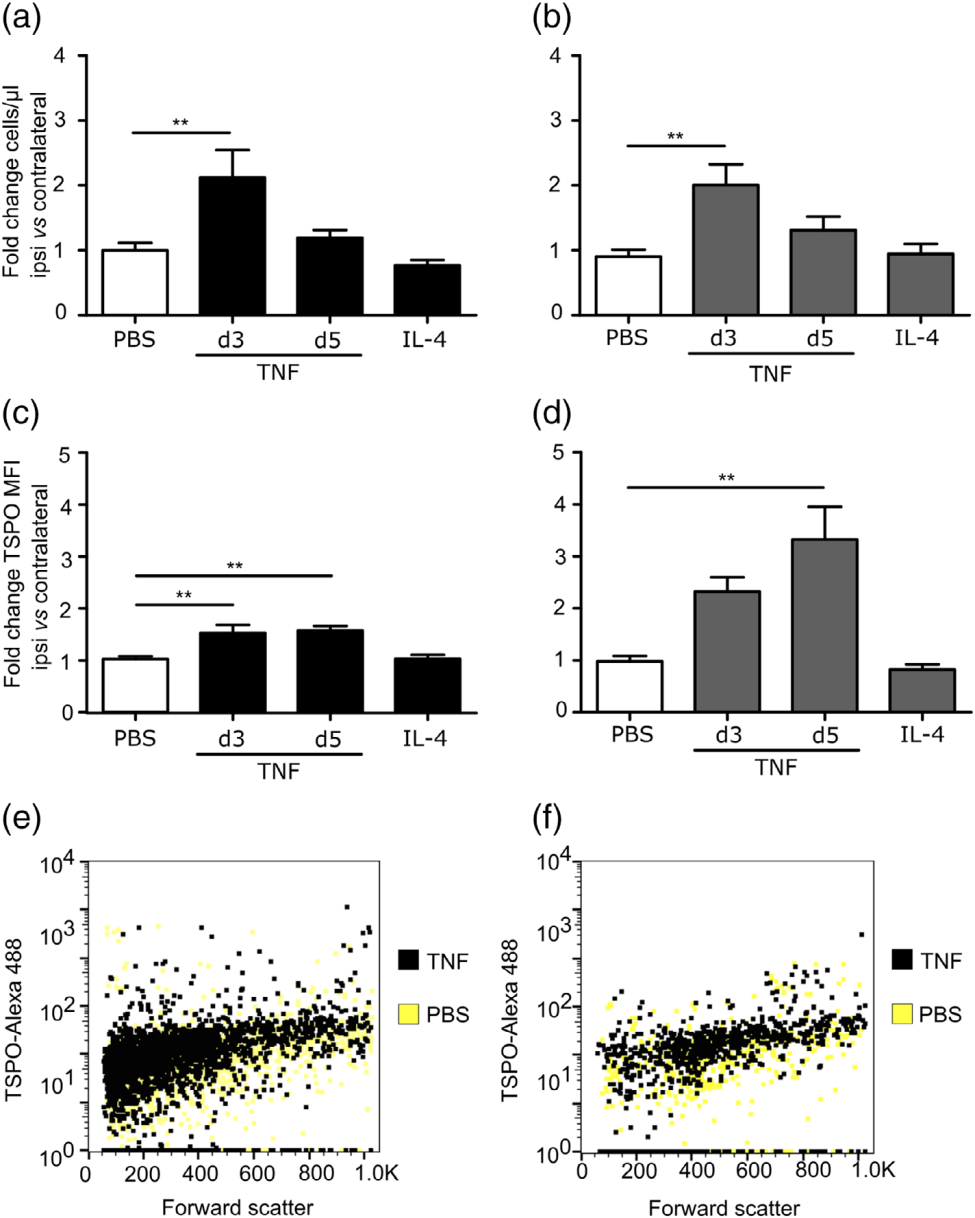
Flow cytometry analysis shows increased number and intensity of staining for TSPO positive astrocytes and microglia after pro‐inflammatory TNF stimulation in vivo. Number of astrocytes (a) and microglia (b) expressing TSPO in ipsilateral hemisphere following injection of AdTNF or IL‐4, normalized to contralateral. Median fluorescence intensity (MFI) of TSPO staining in (c) astrocytes and (d) microglia from ipsilateral hemisphere, normalized to contralateral side (*n* > 6; ANOVA followed by Dunnett's multiple comparison test, *p < .05, **p < .005, ***p < .001). Representative dot blots showing shift in TSPO positive populations of astrocytes (e) and microglia (f), following AdTNF injection compared to PBS injected mice

Representative images of PBS, TNF, and IL‐4 injected animals for PET (a) and autoradiography (b) are shown in Figure 6a,b. A significant increase in TSPO ligand binding was evident in the brains of mice injected with AdTNF compared to PBS (*p* < .001) injected animals (Figure 6c). Similarly, ex vivo autoradiography showed a significant increase in radioactivity in the injected striatum of AdTNF injected mice, compared to PBS injected animals (*p* < .001; Figure 6d). Subsequent immunofluorescent analysis confirmed significantly increased expression of TSPO in astrocytes of AdTNF injected animals compared to PBS injected mice (*p* < .001; Figure 7a–c). Similarly, TSPO expression was significantly increased in microglia/macrophages of AdTNF injected animals compared to PBS injected mice (*p* < .001; Figure 7a,b,d). Subsequent, flow cytometry analysis confirmed these results, showing a significant increase in the number of TSPO positive astrocytes and microglia within the injection site at 3 days after TNF injection (Figure 8a,b) and a significant increase in median fluorescence intensity (MFI) of astrocytes at both Days 3 and 5 after TNF injection. A significant increase in MFI of microglia was also observed 5 days after TNF (Figure 8c,d). No increase in TSPO positive cells or MFI was seen in astrocytes and microglia from IL‐4 injected animals (Figure 8). In order to determine the contribution of endothelial cells to the TSPO expression, we determined the number of TSPO positive endothelial cells, and the MFI of TSPO expression. Although there was a significant increase in the number of TSPO positive endothelial cells in the ipsilateral hemisphere, compared to the contralateral (*p* < .05; Figure S5C) at Day 5 after AdTNF injection, there was no significant increase in MFI (Figure S5D). We also calculated percentages of astrocytes, microglia, and macrophages. We found that astrocytes made up ~35% of total cells, microglia 11%, and endothelial cells 4% with little variation between ipsi versus contralateral and between treatment groups (data not shown). MRI assessment of CBV, which could affect ligand uptake, showed no significant changes in CBV compared to the control contralateral hemisphere in either TNF or IL‐4 injected mice, or compared to PBS injected control animals (Figure S8).

### 3.4 Astrocytes and microglia/macrophages display pro‐ and anti‐inflammatory markers following AdTNF or IL‐4 injection

**Figure 9:**
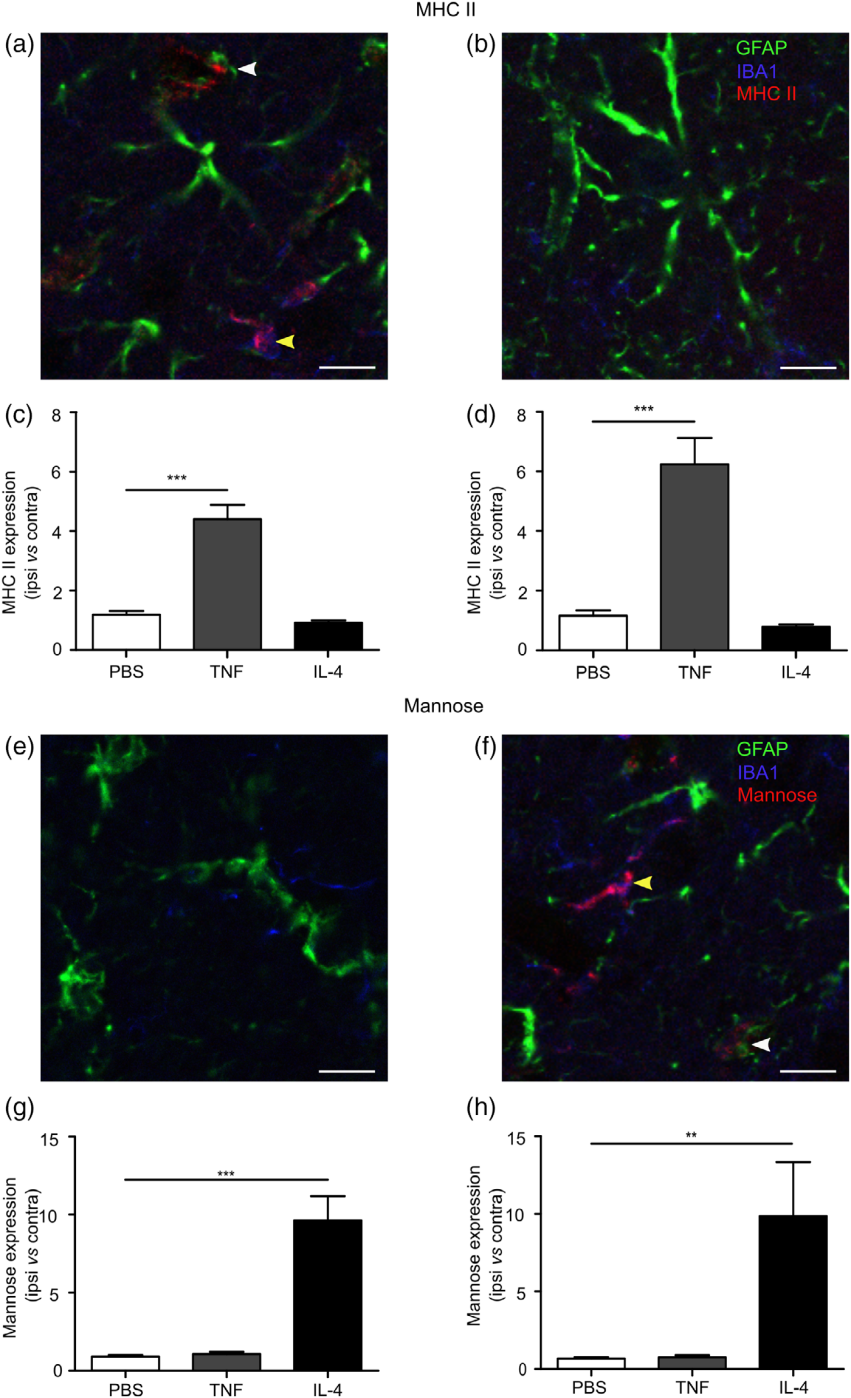
Astrocytes and microglia/macrophages display pro‐ and anti‐inflammatory markers after TNF or IL‐4 stimulation in vivo. (a,b) Representative images of MHC II staining (red) from the injected striatum of an AdTNF injected animal (a) and an IL‐4 injected animal (b). Colocalization with GFAP‐positive astrocytes (green, white arrow) and Iba1 positive microglia/macrophages (blue, yellow arrow) is evident in AdTNF, but not IL‐4, injected brains. (c) Graph showing ratio of MHC II expression in GFAP positive astrocytes in injected (ipsilateral) versus contralateral hemisphere. (d) Graph showing ratio of MHC II expression in Iba1 positive microglia/macrophages in injected (ipsilateral) versus contralateral hemisphere. (e,f) Representative images of CD206 (mannose receptor) staining (red) from the injected striatum of an AdTNF injected animal (e) and an IL‐4 injected animal (f). Colocalization with GFAP‐positive astrocytes (green, white arrow) and Iba1 positive microglia/macrophages (blue, yellow arrow) is evident in IL‐4, but not AdTNF, injected brains. (g) Graph showing ratio of mannose receptor expression in GFAP positive astrocytes in injected (ipsilateral) versus contralateral hemisphere. (h) Graph showing ratio of mannose receptor expression in Iba1 positive microglia/brain macrophages in injected (ipsilateral) versus contralateral hemisphere. (*n* = 3; ANOVA followed by Dunnett's multiple comparison test **p < .005, ***p < .001). Scale bar = 10 μm

**Figure 10:**
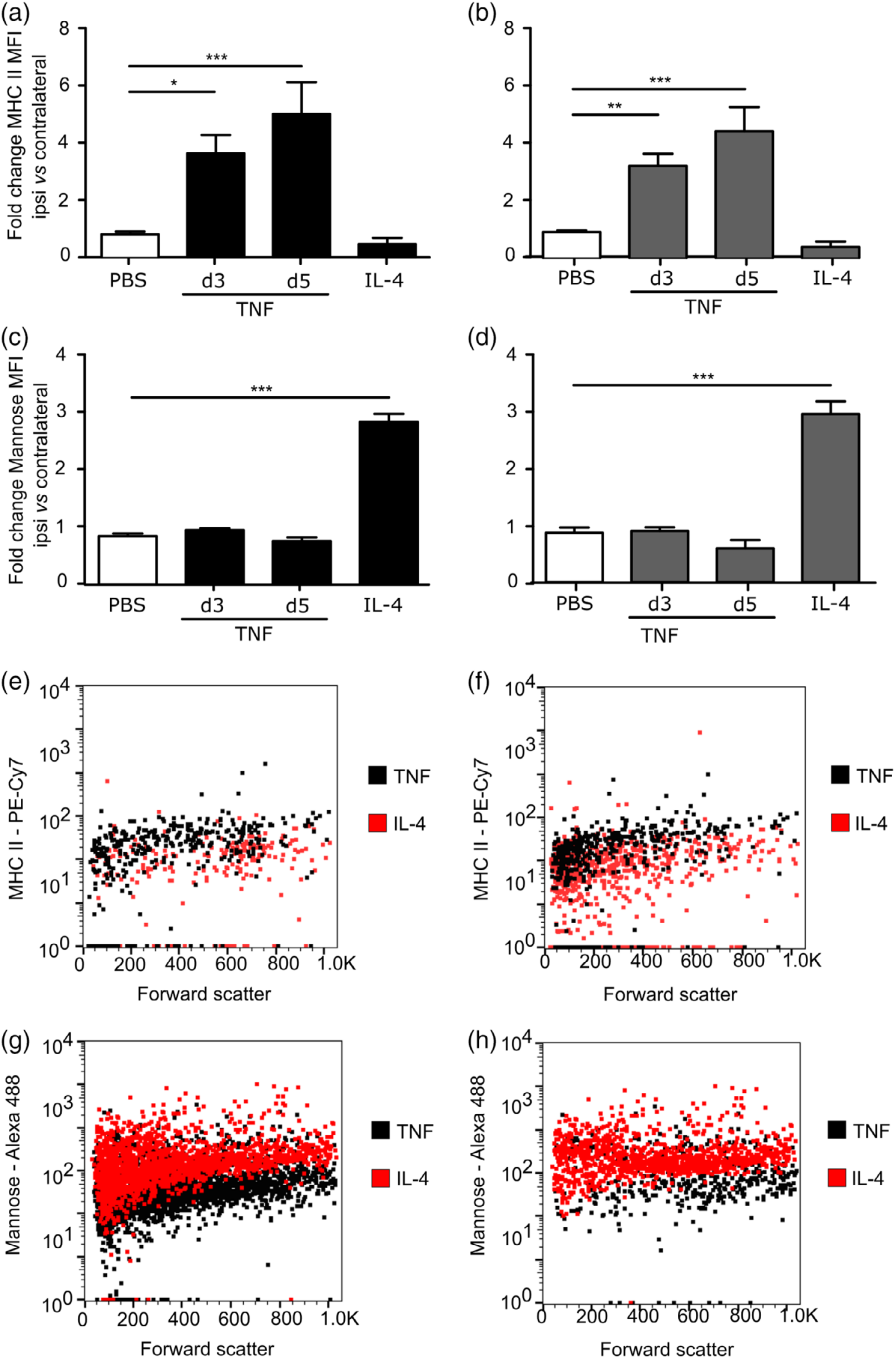
Flow cytometry analysis shows increased MHC II in pro‐inflammatory glia and mannose receptor in anti‐inflammatory glia following TNF or IL‐4 stimulation in vivo. Median Fluorescence Intensity (MFI) of MHC II staining in (a) astrocytes and (b) microglia, and mannose receptor staining in (c) astrocytes and (d) microglia from ipsilateral hemisphere, normalized to contralateral side (*n* > 6; ANOVA followed by Dunnett's multiple comparison test, *p < .05, **p < .005, ***p < .001). Representative dot blots showing shift in MHC II positive populations of astrocytes (e) and microglia (f), following AdTNF injection compared to IL‐4 injected mice, and shift in mannose receptor positive populations of astrocytes (g) and microglia (h), following IL‐4 injection compared to AdTNF injected mice

To confirm that the increase in TSPO expression in astrocytes and microglia/macrophages following TNF stimulation was associated specifically with a pro‐inflammatory phenotype, sections were stained for MHC II as a marker for pro‐inflammatory astrocytes, microglia, and macrophages (Peng, Geil Nickell, Chen, McClain, & Nixon, 2017; Zeinstra et al., 2006) and CD206 (mannose receptor) as an anti‐inflammatory marker (Burudi, Riese, Stahl, & Régnier‐Vigouroux, 1999; Peng et al., 2017). MHC II expression was markedly upregulated in microglia/macrophages and astrocytes in mice injected intrastriatally with AdTNF (Figure 9a), but not those injected with IL‐4 (Figure 9b). Quantitatively, a significant increase in MHC II expressing astrocytes (*p* < .001) and microglia/macrophages (*p* < .001) was evident compared to PBS‐injected injected mice (Figure 9c,d). No significant increase in MHC II expression was found in IL‐4 injected mice. Conversely, no increase in CD206 was found in AdTNF injected mice (Figure 9e,g,h), while 10 fold more CD206 expressing astrocytes (*p* < .001) and microglia/macrophages (*p* < .005) were evident in IL‐4 injected mice compared to PBS injected animals (Figure 9f–h). To further confirm these findings, microglia and astrocytes were isolated from brain tissue following AdTNF or IL‐4 injection and analyzed by flow cytometry for MHC II and mannose receptor expression (Figure 10). Significant upregulation of MHC II expression was evident in both astrocytes and microglia at 3 and 5 days after AdTNF injection, but not IL‐4 injection (Figure 10a,b). Conversely, the anti‐inflammatory mannose receptor was significantly increased in both cell types following IL‐4, but not AdTNF, injection (Figure 10c,d).

## 4 DISCUSSION

The use of TSPO as a biomarker for activated microglia in neurological disease with PET imaging has increased in popularity in recent years (Vivash & O'Brien, 2016). Moreover, recent studies have shown that this biomarker is not restricted to microglia/macrophages, but also reflects activated astrocytes. However, although it is known that the role these cells play in disease depends critically on their inflammatory phenotype, the extent to which TSPO upregulation reflects a pro‐ or anti‐inflammatory phenotype remains unclear. In this study, we have demonstrated that primary mouse astrocytes, microglia, and bone marrow‐derived macrophages polarized to a pro‐inflammatory phenotype show significant TSPO upregulation, which is not seen in anti‐inflammatory polarized cells. Further, utilizing in vivo models of CNS inflammation, we have demonstrated that TSPO expression is only upregulated in microglia/macrophages and astrocytes following induction with a pro‐inflammatory stimulus. Finally, we have demonstrated that the use of a radioligand to TSPO in combination with PET imaging is selective for pro‐inflammatory, and not anti‐inflammatory microglia/macrophage/astrocyte activation.

### 4.1 TSPO upregulation reflects pro‐inflammatory microglia/macrophages and astrocytes specifically

In accord with our findings, Beckers et al. (2018) very recently showed increased microglial TSPO expression in pro‐inflammatory activated microglia in vitro. These authors also demonstrated TSPO upregulation in a transgenic mouse strain that displays widespread neuroinflammation, and which the authors link to a pro‐inflammatory microglial phenotype. However, in a previous study the proliferative microglia from this transgenic strain were shown to display a mixed pro‐ and anti‐ inflammatory phenotype (Verheijden et al., 2015). Consequently, these findings do not provide conclusive in vivo evidence of TSPO expression selectivity for pro‐inflammatory microglia. Nevertheless, in the same study, intracerebroventricular injection of IL‐4 did not induce a change in TSPO expression, which is in accord with our findings following intrastriatal IL‐4 injection. Owen et al. (2017) also recently demonstrated upregulation of TSPO in cultured primary microglia from mice in response to LPS stimulation. Interestingly, in human cultured adult microglia no significant change in TSPO expression was found with either pro‐ or anti‐inflammatory stimulation. In contrast, foetal human microglia did show a trend toward increased TSPO expression with pro‐inflammatory stimulation, although this did not reach significance.

In both of the above studies, however, changes in TSPO expression in astrocytes were not assessed, despite other studies demonstrating that TSPO is not restricted to microglia (Itzhak, Baker, & Norenberg, 1993; Lavisse et al., 2012; O'Brien et al., 2014). Although it is well‐established that microglia/macrophages can exhibit primarily pro‐ or anti‐inflammatory phenotypes, and indeed a spectrum of phenotypes in between, the same distinction has only recently been proposed for astrocytes (Clarke et al., 2018; Liddelow & Barres, 2017). In the current study, we characterized pro‐ and anti‐inflammatory astrocytes, in addition to microglia and macrophages, using the pro‐inflammatory surface marker MHC II and the anti‐inflammatory surface marker CD206 (mannose receptor). Analysis of cultured microglia and macrophages stimulated with LPS and TNF showed a significant upregulation of MHC II expression, however, in cultured astrocytes this was only seen after LPS stimulation. Cultured microglia, macrophages, and astrocytes all showed a significant increase in mannose expression after stimulation with IL‐4. Although mannose receptor expression on astrocytes has been described (Régnier‐Vigouroux, 2003), and has also been shown to be upregulated upon anti‐inflammatory stimuli (Burudi et al., 1999), we also analyzed the expression of CD14 and CLCF1 on pro‐ and anti‐inflammatory astrocytes, which have previously been shown to be upregulated at the mRNA level on IL‐4 stimulated astrocytes using qPCR (Liddelow et al., 2017), We did not, however, find an increase in either marker on IL‐4 stimulated astrocytes using flow cytometry, indicating no change in expression at the protein level. In order to compare our anti‐inflammatory astrocytes with the hypoxia‐induced A2 phenotype in the same paper, we also examined the expression of CD109, which was shown to be upregulated at the mRNA level on A2 astrocytes following middle cerebral artery occlusion. We did not find upregulation of this marker on IL‐4 stimulated astrocytes either in vitro, or in vivo on astrocytes isolated from mouse brain injected with IL‐4 (data not shown). Interestingly, however, this marker was found to be decreased in the LPS‐induced model of pro‐inflammatory activation. These findings suggest that the anti‐inflammatory astrocyte phenotype induced by IL‐4 in vivo differs from the newly described hypoxia‐induced A2 phenotype (Liddelow et al., 2017). These differences in phenotype likely indicate a spectrum of astrocyte activation dependent on stimulus and context, as has been demonstrated for macrophages/microglia (Martinez & Gordon, 2014).

In addition to surface marker analysis, we examined differences in IL‐6, MCP‐1, and RANTES release by primary astrocytes and, depending on the type of stimulus, these data support the concept that it is possible to induce a pro‐inflammatory phenotype in these cells. Interestingly, our data show that astrocytes are more responsive to TNF stimulation than microglia and macrophages, with TNF‐stimulated cells releasing higher amounts of MCP‐1 and RANTES directly after stimulation and for a longer duration. Moreover, it is likely that in the context of the brain in vivo, interactions between microglia/macrophages and astrocytes may lead to greater changes in TSPO expression than are measured in isolated cultured cells, as reported by Owen et al. Consequently, it may be premature to conclude that TSPO expression is not specific to pro‐inflammatory glia in human brain, as suggested by Owen et al., and further studies in human tissue are warranted.

### 4.2 Pro‐inflammatory microglia/macrophages and astrocytes are detected in vivo with TSPO‐targeted PET imaging

Colocalization of TSPO with Iba1 positive microglia/macrophages and GFAP positive astrocytes showed that there was an approximately fivefold increase in TSPO expressing microglia/macrophages and an approximately fourfold increase in TSPO expressing astrocytes in TNF injected animals. Microglia have previously been shown to more highly express TSPO at the mRNA level following stimulation with IFN‐gamma, an alternative pro‐inflammatory stimulus (Daugherty et al., 2013). Nevertheless, it is clear that astrocytes readily express and upregulate TSPO during inflammatory disease, and this is in agreement with previous studies indicating that astrocytes also contribute to TSPO activity in PET imaging (Lavisse et al., 2012; O'Brien et al., 2014). Moreover, flow cytometry analysis indicated a significant increase in the number of microglia and astrocytes expressing TSPO in the injected hemisphere, compared to the contralateral side 3 days after AdTNF injection into the brain. This number decreased at Day 5, but the MFI, or the median expression level of TSPO+ cells, was increased in both cell types at this time point. MFI was also significantly increased in microglia at Day 3. Taken together, these findings suggest that in the initial stages of inflammation, more cells express TSPO, but over time the intensity of expression increases in TSPO+ astrocytes leading to the observed uptake of the TSPO ligand in our PET binding studies.

A recent study by Betlazar, Harrison‐Brown, Middleton, Banati, and Liu (2018) showed that endothelial cells contribute to TSPO expression in the normal brain. In order to determine the contribution of endothelial cells to the TSPO signal in our imaging studies, we used flow cytometry to evaluate endothelial expression. Initial studies in an endothelial cell line showed low TSPO expression, which was downregulated after both pro‐ and anti‐inflammatory stimuli. Similarly, analysis of endothelial cells isolated from mouse brains 3 and 5 days after AdTNF injection showed no significant increase in TSPO expression, although there was a significant increase in the number of endothelial cells expressing TSPO within the injection site compared to the contralateral striatum. However, absolute cell counts of astrocytes, microglia, and endothelial cells showed that the proportion of cells types in the ipsilateral and contralateral samples taken for flow cytometry analysis were consistent at ~35% for astrocytes, 11% for microglia, and 4% for endothelial cells (data not shown). This finding, coupled with the fact that the MFI of TSPO expression on endothelial cells was not significantly increased either in vivo or in vitro, suggests that the contribution of endothelial TSPO in our imaging studies is negligible.

While we have demonstrated a significant contribution of astrocytic TSPO in our pro‐inflammatory neuroinflammation model, it is clear that this contribution varies greatly depending on disease models and astrocytic phenotype. Two imaging studies using the TSPO ligand PK11195 in rat models of cerebral ischemia found that astrocytes did not contribute to the TSPO expression in the lesion (Myers et al., 1991; Stephenson et al., 1995). These studies indicate that TSPO expression on microglia and astrocytes varies in pathology, which is also supported by a study examining TSPO expression on astrocytes and microglia following traumatic brain injury (TBI; Raghavendra Rao, Dogan, Bowen, & Dempsey, 2000). The latter demonstrated that both astrocytes and microglia contributed to [3H]PK11195 binding following TBI in rats, but found that microglia were the more significant contributor to this expression.

Immunofluorescence staining for MHC II and the mannose receptor (CD206), reported as pro‐ and anti‐inflammatory markers, respectively (Burudi et al., 1999; Peng et al., 2017; Zeinstra et al., 2006), confirmed an increase in MHC II positive (pro‐inflammatory) astrocytes and microglia/macrophages following AdTNF injection, but not after either IL‐4 or PBS injection. Conversely, expression of the mannose receptor was upregulated on astrocytes and microglia/macrophages following IL‐4, but not AdTNF injection. These results were further confirmed by flow cytometry analysis of MHC II and mannose expression on astrocytes and microglia isolated from the brain following AdTNF or IL‐4 injection. These findings suggest that the signal arising from ^18^F‐DPA‐713 binding observed exclusively in AdTNF injected animals reflects pro‐inflammatory polarized microglia/macrophages and astrocytes. Critically, therefore, our findings demonstrate not only that both microglia/macrophages and astrocytes exhibit increased TSPO expression specifically when polarized to a pro‐inflammatory phenotype, but that this can be selectively detected in vivo using TSPO‐targeted PET imaging.

In summary, we have demonstrated that TSPO is upregulated in specifically pro‐inflammatory polarized microglia/macrophages and astrocytes, both in vitro and in vivo. We have further shown that PET imaging of microglia/macrophages and astrocytes with the radioligand ^18^F‐DPA‐713 is specific to the pro‐inflammatory sub‐populations of these cells. These findings have significant implications for the use of TSPO‐targeted PET imaging clinically; since the inflammatory phenotype of glial cells is critical to their role in neurological disease, being able to specifically detect the pro‐inflammatory population may greatly enhance the utility and application of TSPO imaging.

## CONFLICT OF INTEREST

The authors declare that they have no conflict of interest.

## Supporting information


**Data S1**: Supporting Information
