# Supplementary material for: Imaging of translocator protein upregulation is selective for pro‐inflammatory polarized astrocytes and microglia
Source: Glia. 2019 Sep 3;68(2):280–97. doi: 10.1002/glia.23716 (PMC6916298; doi:10.1002/glia.23716)
Supplement: Supplementary file 1 — Data S1: Supporting Information [file GLIA-68-280-s001.docx]

## 3’-Fluoro-DPA-713 (FDPA-713): Chemistry

**General procedure A**. Under argon atmosphere, a two-neck flask was charged with THF (5 mL) and *n*-butyllithium (12 mmol, 2.5M in *n*-hexane). At −78 °C a solution of acetonitrile (18 mmol in 10 mL THF) was added drop-wise over a period of 20 minutes. Then, the solution was stirred for an additional hour at −78 °C before the chosen benzoate (6 mmol in 10ml THF) was added over a period of 15 minutes. After one hour the reaction mixture was warmed to −45 °C and the stirring continued until complete consumption of starting material was observed *via* TLC. The reaction mixture was quenched using aqueous HCl (2M, 50 mL), diluted with EtOAc (50 mL) and the layers were separated. The organic layer was washed with brine (4 x 40 mL), dried over MgSO_4_ and concentrated to afford the expected 3‐oxo-3-phenylpropanenitrile derivatives.

**General procedure B**. Potassium carbonate (8 mmol) was added to a reaction vessel charged with the chosen 3‐oxo-3-phenylpropanenitrile derivative (4 mmol) in THF (30 mL). Subsequently, 2-bromodiethylacetamide (6mmol) was added dropwise and the reaction mixture was stirred overnight at room temperature. Then, the reaction mixture was quenched using aqueous HCl (1M, 50 mL), followed by dilution with ethyl acetate (60 mL) and the layers were separated. The organic layer was washed with brine (3 x 50 mL), dried over MgSO_4_ and concentrated *in vacuo*. The crude product was flushed through a short plug of silica using EtOAc/cyclohexane (2/8 to 4/6) to give an oil. The resulting oil was dissolved in ethanol (30 mL), treated with hydrazine monohydrate (8 mmol), glacial acetic acid (8 mmol) and heated to reflux for 18 hours. Afterwards the solvent was evaporated under reduced pressure and diluted with ethyl acetate before being washed with saturated sodium carbonate (3 x 50 mL). The organic layer was dried over MgSO_4_, concentrated i*n vacuo* and purified by flash column chromatography (SiO_2_, methanol/ dichloromethane (3/97)) to afford the expected pyrazolo[1,5‐*a*]pyrimidin‐3‐yl]acetamide derivatives.

**General procedure C**. 2,4-Pentanedione (4 mmol) was added to a solution of the chosen pyrazolo[1,5‐*a*]pyrimidin‐3‐yl]acetamide derivative (2 mmol in 10 mL of EtOH). Then, the reaction mixture was heated at reflux for 20 h. Upon cooling to ambient temperature, the solvent was removed in *vacuo*. The crude material was purified by column chromatography (SiO_2_, methanol / dichloromethane (9:1)) to give the expected DPA-713 analogues.

**3‐(3‐Fluoro‐4‐methoxyphenyl)‐3‐oxopropanenitrile.**

Synthesized following general procedure A, yielding 3‐(3‐fluoro‐4‐methoxyphenyl)‐3‐oxopropanenitrile as a white solid (1.14 g, 98%, 95% purity, processed to the next step without further purifications). **Mp** 113 ̶ 115°C; **^1^H NMR** (400 MHz, CDCl_3_) δ 7.69 (ddd, *J* = 8.4, 2.2, 1.0 Hz, 1H), 7.66 (dd, *J* = 11.4, 2.2 Hz, 1H), 7.04 (t, *J* = 8.3 Hz, 1H), 4.03 (s, 2H), 3.98 (s, 3H); **^13^C NMR** (101 MHz, CDCl_3_) δ 185.0, 153.5 (d, *J* = 16 Hz), 152.2 (d, *J* = 247 Hz), 127.5 (d, *J* = 5 Hz), 126.3 (d, *J* = 3 Hz), 116.2 (d, *J* = 20 Hz), 113.9, 112.8, 56.6, 29.3; **^19^F NMR** (376 MHz, CDCl_3_) δ − 132.7 (1F, m); **IR** *ν* 2946, 2259, 1689, 1465, 1286, 1123, 1007, 866, 842, 762, 723; **HRMS** (ESI) for C_10_H_7_FNO_2_ [M-H]^−^ requires 192.0466 found 192.0465.

**2‐[5‐Amino‐3‐(3‐fluoro‐4‐methoxyphenyl)‐*1H*‐pyrazol‐4‐yl]‐*N*,*N*‐diethylacetamide.**

Synthesized following general procedure B, yielding 2‐[5‐amino‐3‐(3‐fluoro‐4‐methoxyphenyl)‐*1H*‐pyrazol‐4‐yl]‐*N*,*N*‐diethylacetamide (0.768 g, 60%) as yellow powder. **Mp** 89 ̶ 90 °C. **^1^H NMR** (400 MHz, CD_3_CN) δ 7.24-7.15 (m, 2H), 7.13-7.08 (m, 1H), 4.18 (s, 2H), 3.87 (s, 3H), 3.43 (s, 2H), 3.29 (q, *J* = 7.1 Hz, 2H), 3.18 (q, *J* = 7.1 Hz, 2H), 1.02 (t, *J* = 7.1 Hz, 3H), 0.98 (t, *J* = 7.1 Hz, 3H) (Signal of pyrrazole NH is not observed); **^13^C NMR** (101 MHz, CD_3_CN) δ 171.0, 152.9 (d, *J* = 244 Hz), 148.4 (d, *J* = 11 Hz), 124.8 (d, *J* = 3 Hz), 118.3 (4C), 116.0 (d, *J* = 19 Hz), 114.7 (d, *J* = 2 Hz), 56.9, 43.0, 41.1, 28.9, 14.4, 13.4; **^19^F** **NMR** (376 MHz, CDCl_3_) δ − 134.1 (1F, t, *J* = 10.0 Hz); **IR** *ν* 3211, 2980, 2934, 1621, 1523, 1380, 1256, 1096, 948, 789; **HRMS** (ESI) for C_16_H_21_FN_4_O_2_ [M-H]^-^ requires 319.1576 found 319.1561.

***N*,*N*‐Diethyl‐2‐[2‐(3‐fluoro‐4‐methoxyphenyl)‐5,7‐dimethylpyrazolo[1,5‐*a*]pyrimidin‐3‐yl]acetamide.**

Synthesized following general procedure C, yielding *N*,*N*‐diethyl‐2‐[2‐(3‐fluoro‐4‐methoxyphenyl)‐5,7‐dimethylpyrazolo[1,5‐*a*]pyrimidin‐3‐yl]acetamide. yield (0.69 g, 90%) as a yellow powder. **Mp** 100 ̶ 101 °C; **^1^H NMR** (400 MHz, CDCl_3_) δ 7.65-7.60 (m, 2H), 7.03 (t, *J* = 8.4 Hz, 1H), 6.51 (d, *J* = 0.8 Hz, 1H), 3.92 (s, 3H), 3.92 (s, 2H), 3.53 (q, *J* = 7.1 Hz, 2H), 3.41 (q, *J* = 7.1 Hz, 2H), 2.73 (d, *J* = 0.8 Hz, 3H), 2.53 (s, 3H), 1.23 (t, *J* = 7.1 Hz, 3H), 1.11 (t, *J* = 7.1 Hz, 3H); **^13^C NMR** (101 MHz, CDCl_3_) δ 170.1, 157.8, 153.9, 152.6 (d, *J* = 245 Hz), 148.0 (d, *J* = 2 Hz), 147.9 (d, *J* = 6 Hz), 144.9, 127.2 (d, *J* = 6 Hz), 124.8 (d, *J* = 4 Hz), 116.5 (d, *J* = 20 Hz), 113.4, 108.6, 101.1, 56.5, 42.5, 40.8, 28.2, 24.8, 17.0, 14.5, 13.2; **^19^F NMR** (376 MHz, CDCl_3_) δ − 135.4 (1F, m); **IR** *ν* 2971, 1641, 1442, 1271, 1181, 1026, 852, 786, 762, 649; **HRMS** (ESI) for C_21_H_24_FN_4_O_2_ [M-H]^-^ requires 383.1888 found 383.1863.

**3‐(3‐Bromo‐4‐methoxyphenyl)‐3‐oxopropanenitrile.**

Synthesized following general procedure A, yielding 3‐(3‐bromo‐4‐methoxyphenyl)‐3‐oxopropanenitrile (1.42 g, 93%) as yellow powder. **Mp** 110 ̶ 112 °C; **^1^H NMR** (400 MHz, CDCl_3_) δ 8.33 (s, 1H), 7.92 (d, *J* = 8.7 Hz, 1H), 6.89 (d, *J* = 8.7 Hz, 1H), 4.02 (s, 2H), 3.98 (s, 3H); **^13^C NMR** (101 MHz, CDCl_3_) δ 184.5, 163.1, 140.3, 131.0, 128.9, 113.8, 110.5, 86.6, 57.0, 29.2. **IR** *ν* 980, 2915, 2255, 1687, 1589, 1285, 1202, 966, 828, 818, 774, 676; **HRMS** (ESI) for C_10_H_7_O_2_N^79^Br [M-H]^-^ requires 251.9665 found 251.9674.

**2‐[5‐Amino‐3‐(3‐bromo‐4‐methoxyphenyl)‐*1H*‐pyrazol‐4‐yl]‐*N*,*N*‐diethylacetamide.**

Synthesized following general procedure C, yielding 2‐[5‐amino‐3‐(3‐bromo‐4‐methoxyphenyl)‐*1H*‐pyrazol‐4‐yl]‐*N*,*N*‐diethylacetamide (0.81 g, 53%) as a yellow powder. **Mp** 84 ̶ 86 °C; **^1^H NMR** (400 MHz, CDCl_3_) δ 7.52 (d, *J* = 2.1 Hz, 1H), 7.26 (dd, *J* = 8.5, 2.1 Hz, 1H), 6.82 (d, *J* = 8.5 Hz, 1H), 3.88 (s, 3H), 3.44 (s, 2H), 3.31 (q, *J* = 7.1 Hz, 2H), 3.09 (q, *J* = 7.1 Hz, 2H), 1.07 (t, *J* = 7.1 Hz, 3H), 0.95 (t, *J* = 7.1 Hz, 3H) (Signal due to pyrrazole NH and NH_2_ are not observed); **^13^C NMR** (101 MHz, CDCl_3_) δ 170.3, 155.7, 153.4, 142.3, 142.3, 132.4, 125.1, 111.9, 111.7, 97.2, 56.3, 42.4, 40.6, 28.4, 14.2, 14.1; **IR** *ν* 3542, 2915, 1615, 1595, 1469, 1428, 1229, 1107, 971, 832, 747; **HRMS** (ESI) for C_16_H_21_^79^BrN_4_O_2_ [M-H]^-^ requires 379.0775, found 379.0770.

***N*,*N*‐Diethyl‐2‐[2‐(3‐bromo‐4‐methoxyphenyl)‐5,7‐dimethylpyrazolo[1,5‐*a*]pyrimidin‐3‐yl]acetamide.**

Synthesised following general procedure C, yielding *N*,*N*‐diethyl‐2‐[2‐(3‐bromo‐4‐methoxyphenyl)‐5,7‐dimethylpyrazolo[1,5‐*a*]pyrimidin‐3‐yl]acetamide (0.80 g, 90%) as a yellow powder. **Mp** 145 ̶ 146 °C; **^1^H NMR** (400 MHz, CDCl_3_) δ 7.99 (d, *J* = 2.1 Hz, 1H), 7.84 (dd, *J* = 8.5, 2.1 Hz, 1H), 6.97 (d, *J* = 8.5Hz, 1H), 6.51 (d, *J* = 0.9 Hz, 1H), 3.93 (s, 3H), 3.91 (s, 2H), 3.52 (q, *J* = 7.1 Hz, 2H), 3.41 (q, *J* = 7.1 Hz, 2H), 2.73 (d, *J* = 0.8 Hz, 4H), 2.53 (s, 3H), 1.23 (t, *J* = 7.1 Hz, 3H), 1.12 (t, *J* = 7.1 Hz, 3H); **^13^C NMR** (101 MHz, CDCl_3_) δ 169.9, 157.8, 156.0, 153.6, 147.9, 144.9, 133.3, 129.1, 127.9, 111.9, 111.8, 108.5, 101.1, 56.4, 42.5, 40.8, 28.1, 24.8, 17.0, 14.5, 13.2; **IR** *ν* 2980, 1638, 1554, 1225, 1052, 945, 907, 845, 787, 763; **HRMS** (ESI) for C_21_H_24_BrN_4_O_2_ [M-H]^-^ requires 443.1088 found 443.1081.

***N,N*‐Diethyl‐2‐{2‐[4‐methoxy‐3‐(tetramethyl‐1,3,2‐dioxaborolan‐2‐yl)phenyl]‐5,7‐dimethylpyrazolo [1,5‐*a*]pyrimidin‐3‐yl}acetamide.**

Under argon atmosphere, a round-bottomed flask was charged with toluene (2 mL) followed by addition of *N*,*N*‐diethyl‐2‐[2‐(3‐bromo‐4‐methoxyphenyl)‐5,7‐dimethylpyrazolo[1,5‐*a*]pyrimidin‐3‐yl]acetamide (177 mg, 0.4 mmol), Pd(dppf)Cl_2_.CH_2_Cl_2_ (32 mg, 0.04 mmol), potassium acetate (157 mg, 1.6 mmol) and bis(pinacolato)diboron (305 mg, 1.2 mmol). Then the reaction mixture was stirred at 85 °C for 18 hours. Afterwards the reaction mixture was allowed to cool to ambient temperature and the solvent removed in *vacuo.* After addition of ethyl acetate (5 mL) the mixture was filtered through a short plug of celite and concentrated in *vacuo*. The crude product was first purified by flash column chromatography (SiO_2_, acetone/dichloromethane (1/9 to 2/8)) followed by recrystallization from petroleum ether and diethylether to afford *N,N*‐diethyl‐2‐{2‐[4‐methoxy‐3‐(tetramethyl‐1,3,2‐dioxaborolan‐2‐yl)phenyl]‐5,7‐dimethylpyrazolo[1,5‐*a*]pyrimidin‐3‐yl}acetamide as a white powder (118mg, 60%). **Mp** 120 ̶ 122 °C; **^1^H NMR** (400 MHz, CDCl_3_) δ 8.02 (d, *J* = 2.3 Hz, 1H), 7.88 (dd, *J* = 8.6, 2.3 Hz, 1H), 6.93 (d, *J* = 8.6 Hz, 1H), 6.48 (d, *J* = 0.7 Hz,1H), 3.89 (s, 2H), 3.85 (s, 3H) (3.46 (q, *J* = 7.2 Hz, 2H), 3.39 (q, *J* = 7.1 Hz, 2H), 2.74 (d, *J* = 0.7 Hz, 3H), 2.52 (s, 3H), 1.36 (s, 12H), 1.18 (t, *J* = 7.2 Hz, 3H), 1.10 (t, *J* = 7.1 Hz, 3H); **^13^C NMR** (101 MHz, CDCl_3_) δ 170.1, 164.6, 157.5, 155.2, 147.8, 144.8, 137.0, 133.3, 126.0, 110.6, 108.2, 101.0, 83.6 (2C), 56.1, 42.4, 40.8, 28.3, 24.9 (4C), 24.8, 17.1, 14.5, 13.3 Signal due to carbon bearing the boron is not observed; **IR** *ν* 2974, 1649, 1585, 1398, 1249, 1140, 1067, 1024, 859, 759; **HRMS** (ESI) for C_27_H_38_^11^BN_4_O_4_ [M+H]^+^ requires 493.2978 found 493.2978.

## 3’-[^18^F]Fluoro-FDPA-713: Radiochemistry: General Experimental Details

^18^F-Fluoride was produced by Alliance Medical (UK) via the ^18^O(p,n)^18^F reaction and delivered as ^18^F-fluoride in ^18^O-water. Radiosynthesis and azeotropic drying was performed on a NanoTek® automated microfluidic device (Advion). Radio-TLC was performed on Merck Kiesegel 60 F254 plates. Analysis was performed using a plastic scintillator/PMT dete ctor. HPLC analysis was performed with a Dionex Ultimate 3000 dual channel HPLC system equipped with shared autosampler, parallel UV-detectors and LabLogic NaI/PMT-radiodetectors with Flowram analog output.

**Synthesis of [Cu(OTf)_2_(impy)_4_] complex**

A solution of imidazo[1,2-b]pyridazine (impy) (758 mg, 6.36 mmol) in MeOH (1 mL) was added dropwise to a solution of Cu(OTf)_2_ (230 mg, 0.636 mmol) in MeOH (1 mL) at 55 °C. After 5 minutes the blue precipitate was filtered off and the resulting crystals washed with Et_2_O (3 x 2 mL). Finally the crude product was purified by recrystallisation from hot MeOH to afford [Cu(OTf)_2_(impy)_4_] (324 mg, 0.387 mmol, 61%) as a blue solid.

Anal. Calcd. for C_26_H_20_CuF_6_N_12_O_6_S_2_: C, 37.26; H, 2.41; N, 20.05. Found: C, 37.07; H, 2.33; N, 19.91; IR (ATR, neat): ν (cm^-1^) = 2981, 1620, 1541, 1503, 1374, 1352, 1306, 1281, 1241, 1221, 1149, 1071, 1027, 950, 918, 879, 801, 755, 733, 632.

**Procedure for the Synthesis and Isolation of 3’-[^18^F]Fluoro-DPA-713:**

**Supplementary Scheme 1:** Synthesis of 3’-[^18^F]Fluoro-DPA-713

[^18^F]Fluoride was separated from ^18^O-enriched-water using an anion exchange cartridge (Sep-Pak Accell Plus QMA Carbonate Plus Light Cartridge, 46 mg Sorbent per Cartridge, 40 µm particle size, Waters) and released with 900 μL (in 6 x 150 µL portions) of a solution of K_222_/K_2_C_2_O_4_/K_2_CO_3_ (kryptofix 222 (6.3 mg), K_2_C_2_O_4_ (1 mg) and K_2_CO_3_ (0.1 mg) in 1 mL of MeCN/H_2_O, 4:1) into a 5 mL V-vial containing a magnetic stir bar in the concentrator. The solution was dried with five cycles of azeotropic drying with MeCN (5 x 200 μL) under a flow of N_2_ at 105 °C. Afterwards the V-vial containing the dried [^18^F]KF/K_2.2.2_ complex was purged with 30 mL of air using a syringe and then a solution of arylboronate precursor (9.9 mg, 0.02 mmol) and Cu(OTf)_2_(impy)_4_ (25 mg, 0.02 mmol) in anhydrous 1,3-dimethyl-2-imidazolidinone (DMI) (300 μL) was added. The mixture was stirred for 20 minutes at 120 °C. The reaction was then cooled to room temperature before diluting with a 1 mL 50% MeCN/ 50% 25 mM aqueous NH_4_HCO_2_ solution and loaded directly onto a 2 mL HPLC loop and injected on a semi-Prep HPLC column (Synergi 4µm Hydro-RP 250 x 10mm, 50% MeCN/ 50% 25 mM aqueous NH_4_HCO_2_, 3 mL/min).

The 3’-[^18^F]Fluoro-DPA-713 was collected in 20 mL of H_2_O and eluted over an Oasis HLB Plus cartridge (preconditioned with 2 mL MeOH and 10 mL H_2_O). The product was then eluted with EtOH (2.0 mL). The ethanol was evaporated under a flow of N_2_ while heating at 120 °C. The dry residue was then re-dissolved in 10% DMSO/PBS (pH = 7.4).

The Molar Activity of 3’-[^18^F]Fluoro-DPA-713 was assessed by radio-HPLC, using an analytical Synergi 4 µm Hydro-RP 80A column, 150 x 4.6 mm eluted with 50% MeCN/50% H_2_O (isocratic 1 mL/min), monitoring with UV (220 nm) and radioactive traces.

**Supplementary Table 1.** 3’-[^18^F]Fluoro-DPA-713 Isolation Results:

| **Run** | **Ligand^a^/**  **Solvent** | **Activity (MBq)** | | | | **AY (%) (n.d.c.)** | **Synthesis Time (min)** | |
| --- | --- | --- | --- | --- | --- | --- | --- | --- |
|  |  | Starting | Dried | Isolated |  |  |  |  |
| 1 | Pyridine/DMA^b^ | 6230 | 5450 | 237 |  | 3.8 | 112 | |
| 2 | Pyridine/DMA^b^ | 6810 | 5810 | 684 |  | 10.0 | 102 | |
| 2 | Pyridine/DMA^b^ | 8190 | 5940 | 203 |  | 2.5 | 117 | |
| **Activity Yield: 5% ± 3% (*n* = 3)** | | | | | | | | |
| 2 | IMPY^c^/DMI^d^ | 3770 | 2990 | 120 |  | 3.2 | 105 | |
| 2 | IMPY^c^/DMI^d^ | 9470 | 7640 | 699 |  | 7.4 | 117 | |
| 3 | IMPY^c^/DMI^d^ | 7030 | 6050 | 462 |  | 6.8 | 109 | |
| **Activity Yield: 6% ± 3% (*n* = 3)** | | | | | | | |  |

^a^Cu(L)_4_(OTf)_2_ where L: Ligand ^b^ DMA: *N,N-*Dimethylacetamide ^c^IMPY: Imidazo[1,2-*b*]pyridazine ^d^DMI: 1,3,-dimethyl-2-imidazolidinone


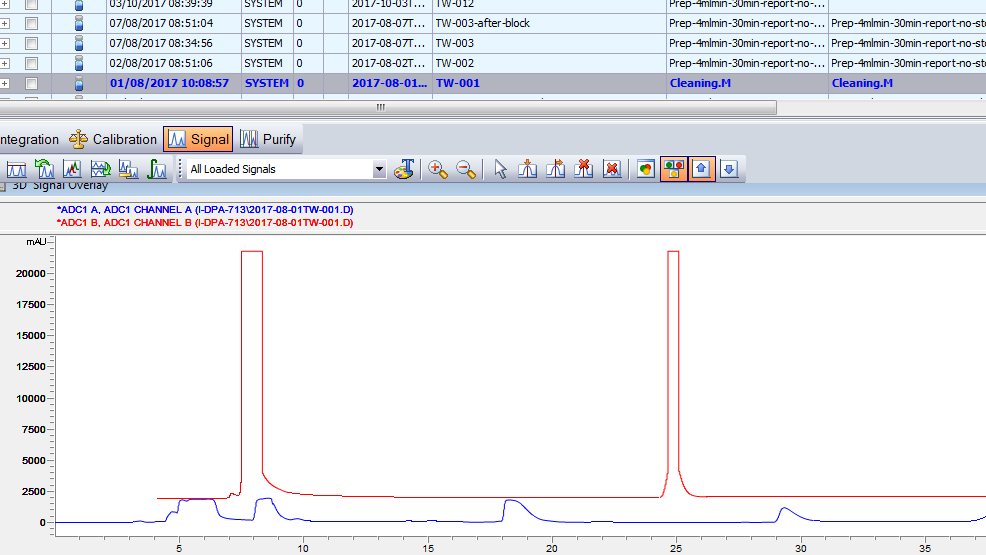


**Supplementary Figure 1.** Semi-prep radioHPLC purification of 3’-[^18^F]Fluoro-DPA-713 (red line); Semi-prep UV trace monitoring at 254 nm (blue line)

**Supplementary Figure 2.** Purified 3’-[^18^F]Fluoro-DPA-713 was injected onto an analytical column. Additionally, an authentic reference sample of 3’-Fluoro-DPA-713 was analysed. Analytical HPLC conditions are listed in the previous section.

**Supplementary Figure 3.** Molar Activity Curve for 3’-[^18^F]Fluoro-DPA-713

**Supplementary Figure 4.** Spectra of Novel Compounds:

**A.** 3‐(3‐Fluoro‐4‐methoxyphenyl)‐3‐oxopropanenitrile


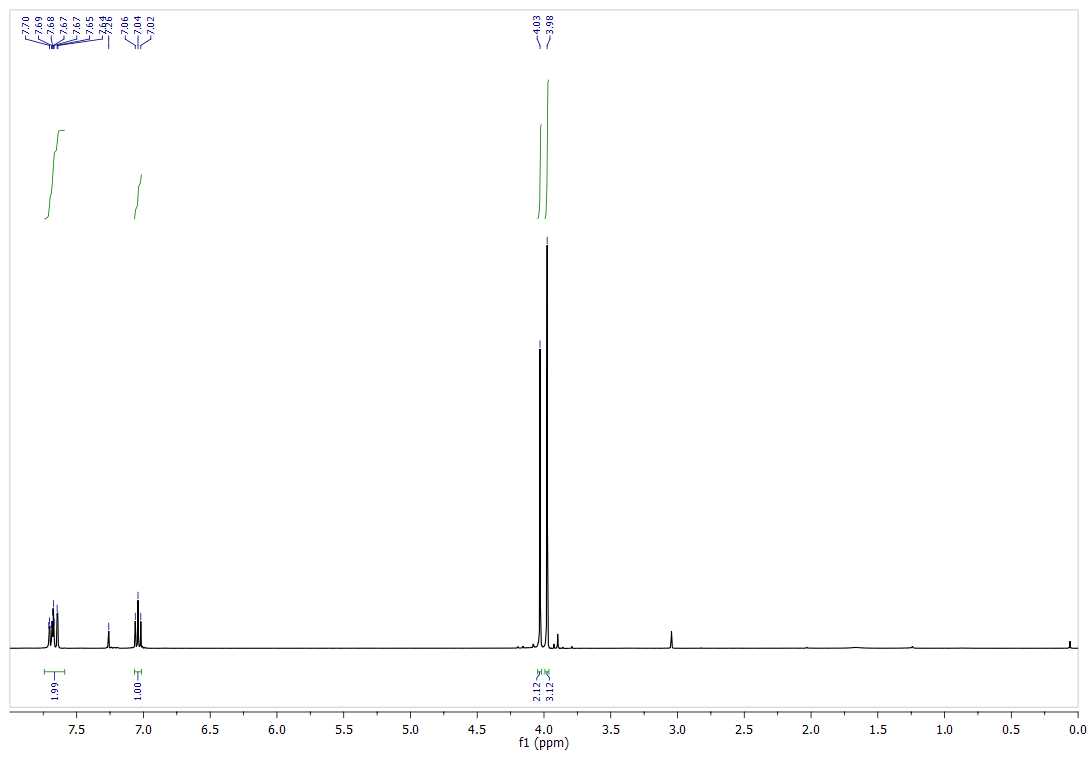


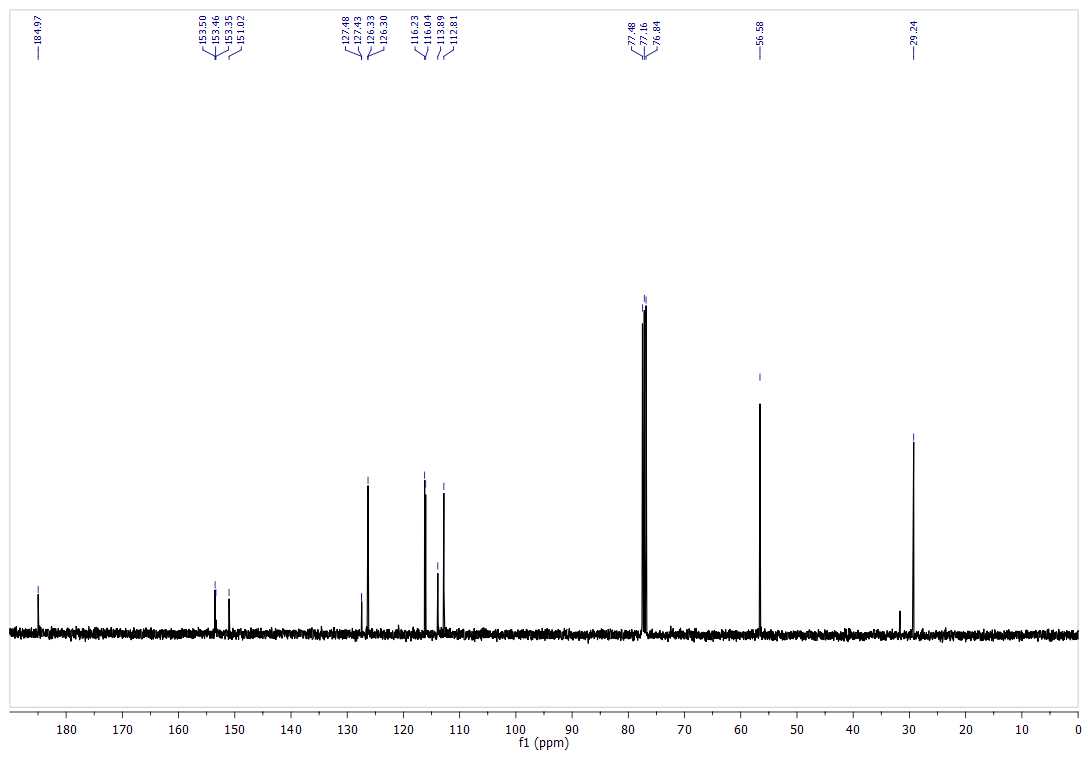

**B.** 2‐[5‐Amino‐3‐(3‐fluoro‐4‐methoxyphenyl)‐*1H*‐pyrazol‐4‐yl]‐N,N‐diethylacetamide


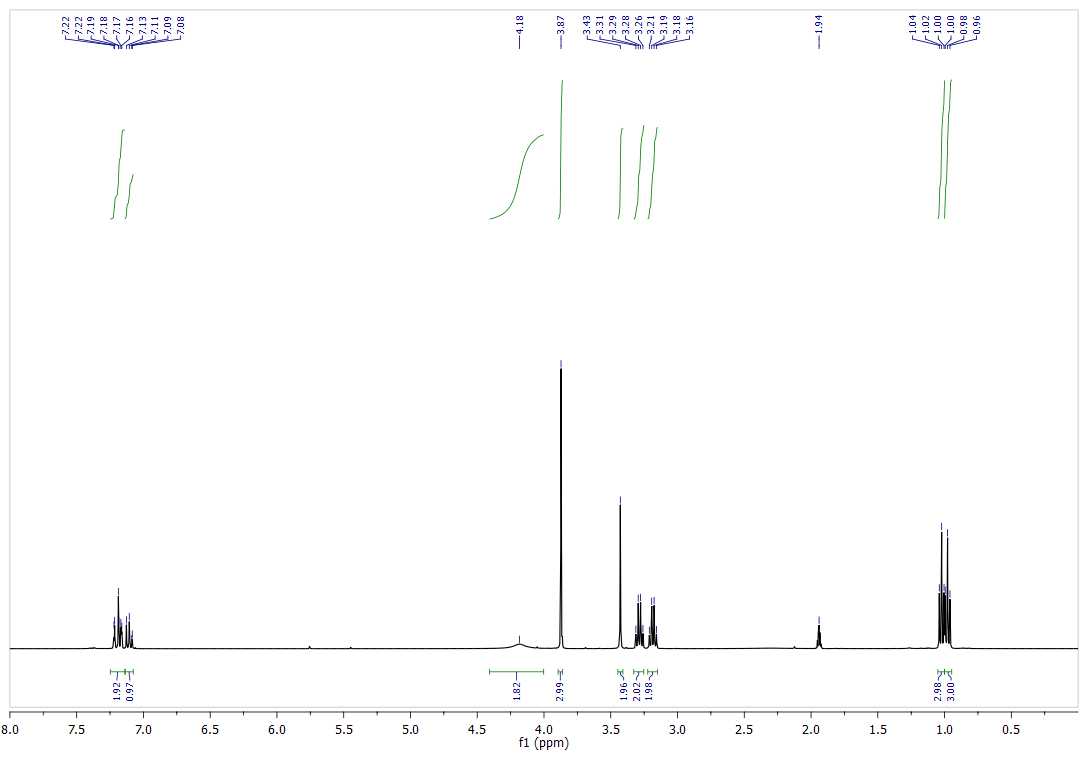


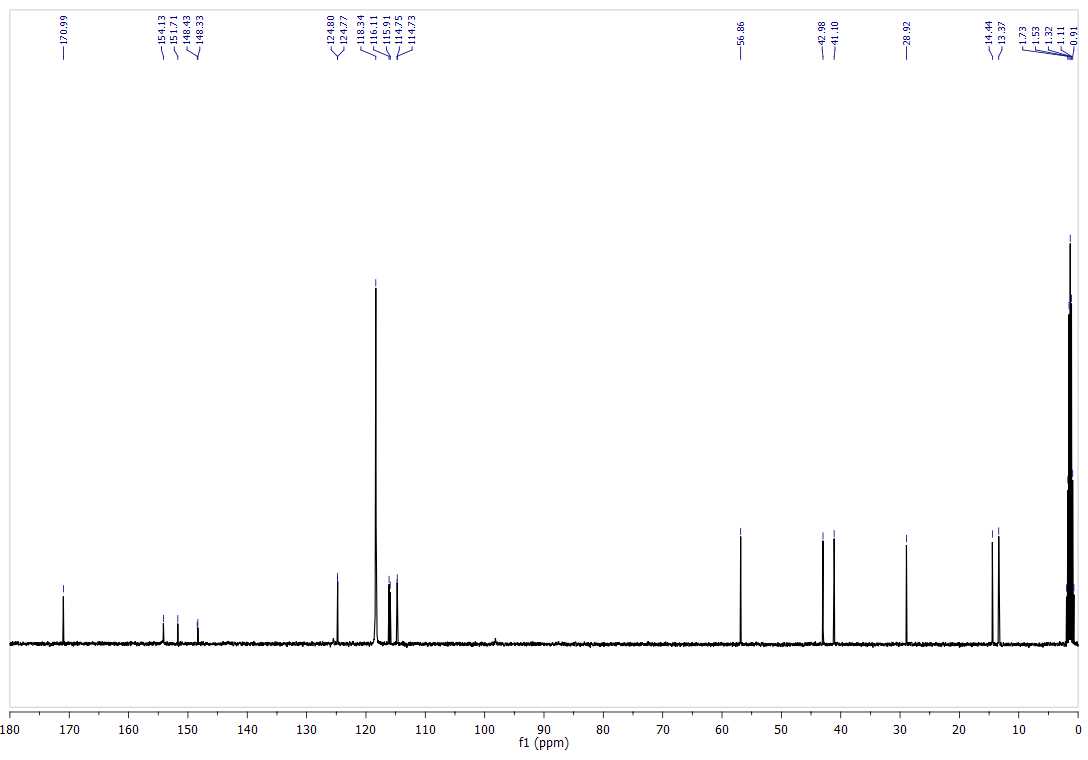

**C.** *N*,*N*‐Diethyl‐2‐[2‐(3‐fluoro‐4‐methoxyphenyl)‐5,7‐dimethylpyrazolo[1,5‐*a*]pyrimidin‐3‐yl]acetamide


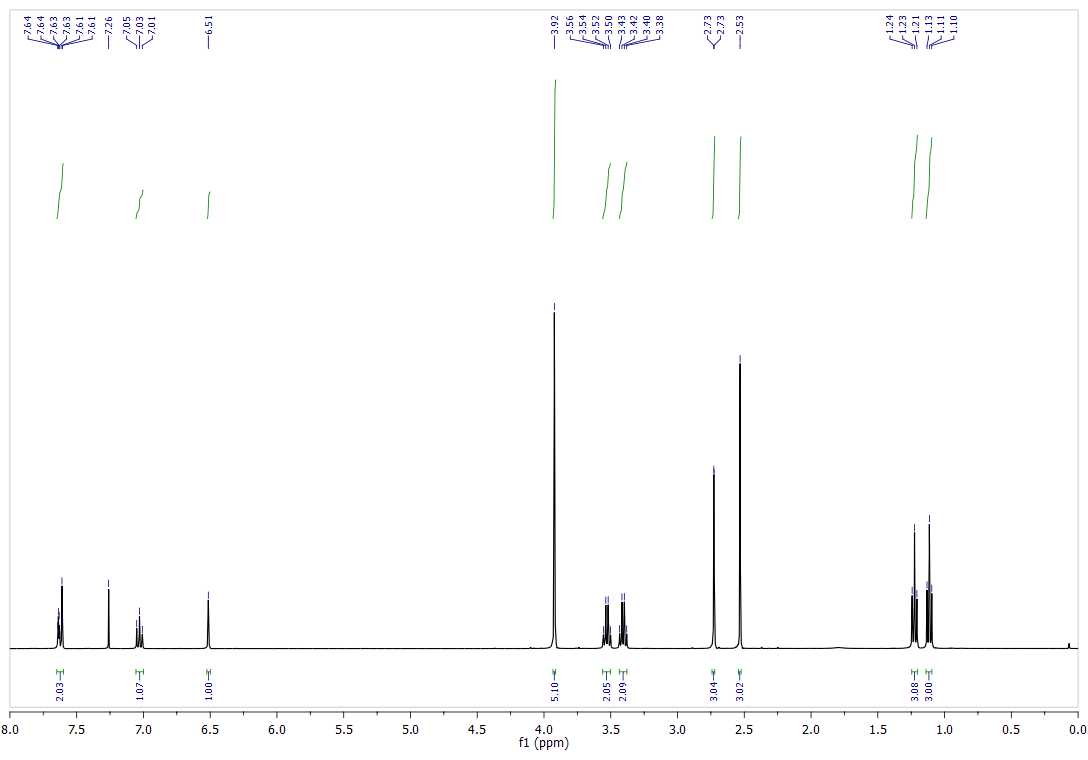


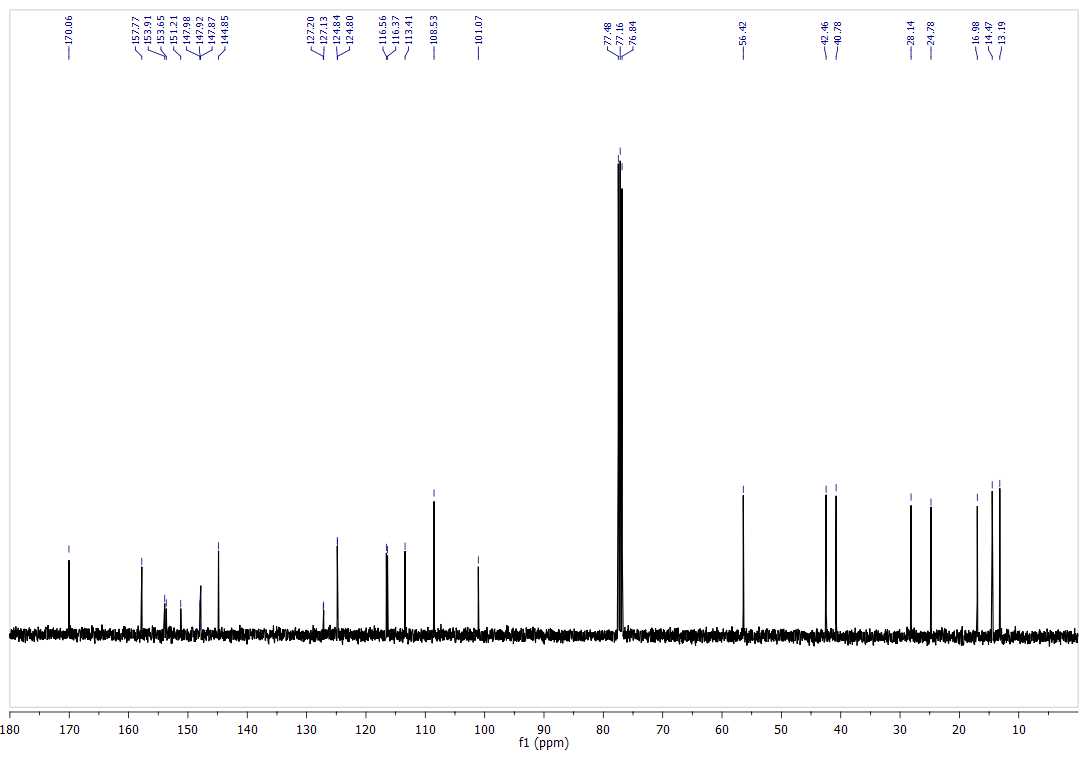

**D.** 3‐(3‐Bromo‐4‐methoxyphenyl)‐3‐oxopropanenitrile


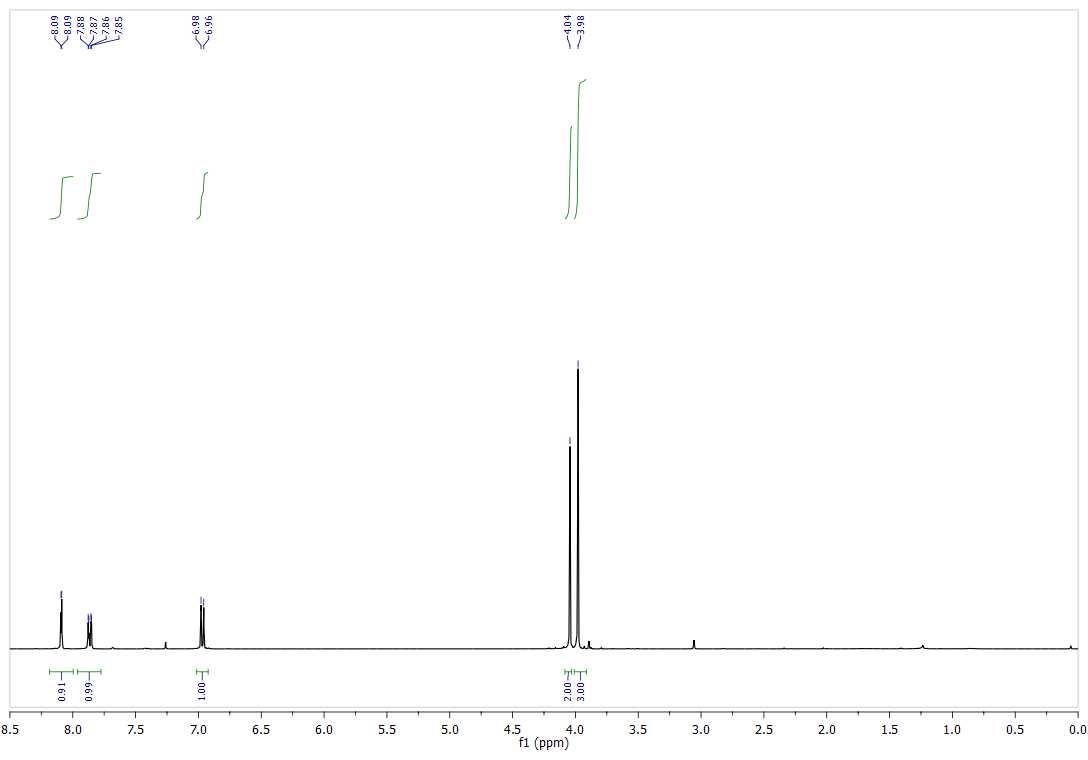


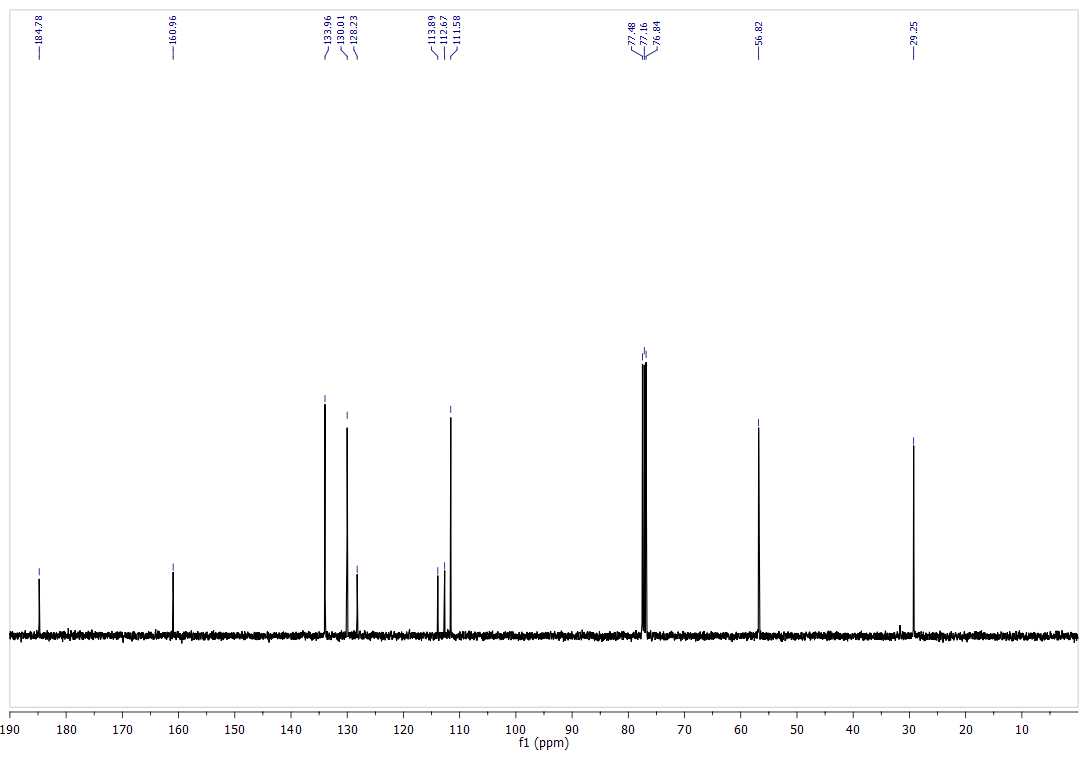


**E.** 2‐[5‐Amino‐3‐(3‐bromo‐4‐methoxyphenyl)‐*1H*‐pyrazol‐4‐yl]‐N,N‐diethylacetamide


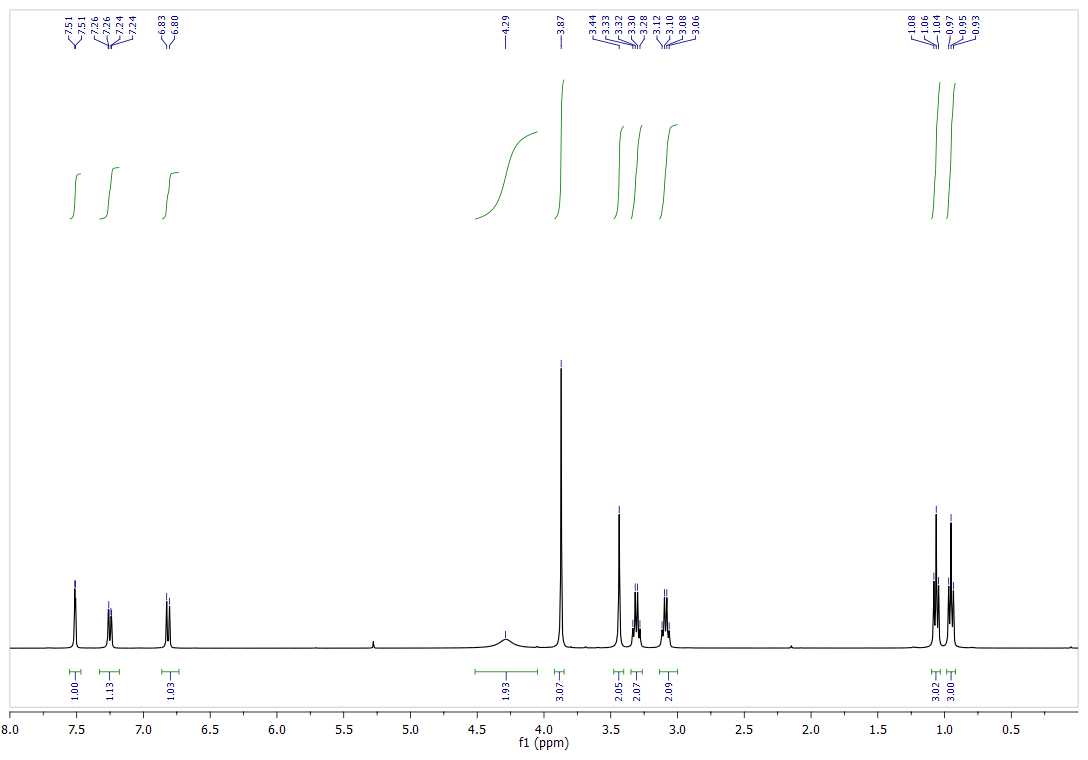


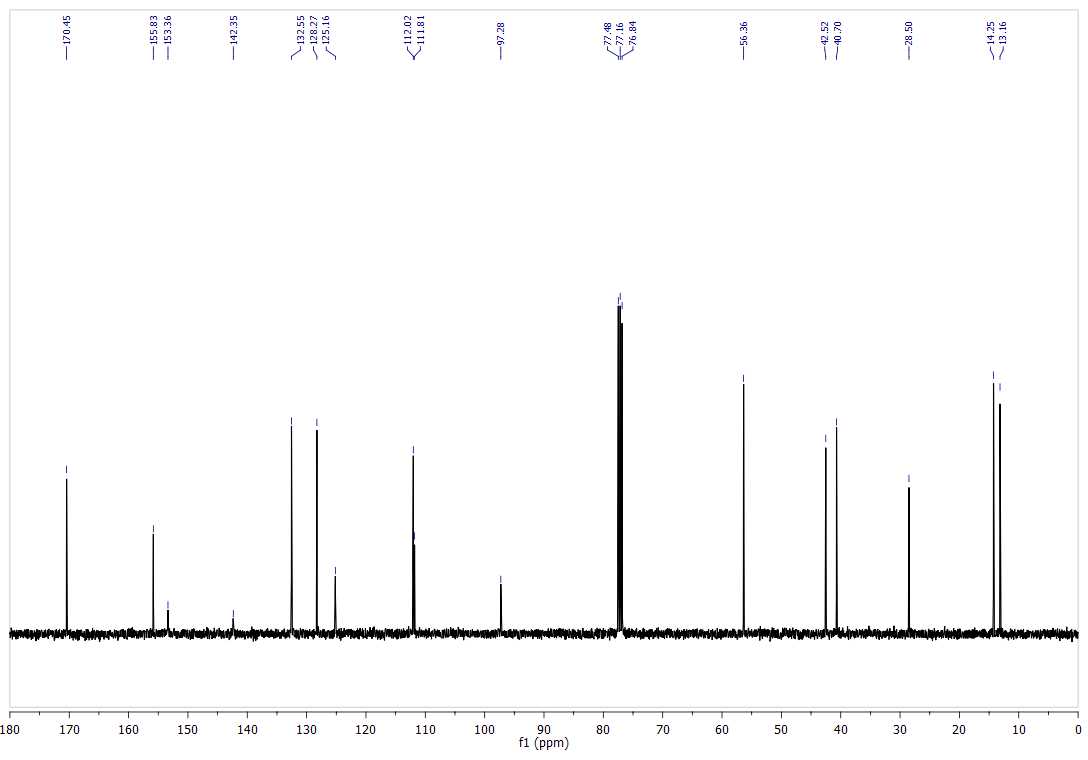


**F.** *N*,*N*‐Diethyl‐2‐[2‐(3‐bromo‐4‐methoxyphenyl)‐5,7‐dimethylpyrazolo[1,5‐*a*]pyrimidin‐3‐yl]acetamide


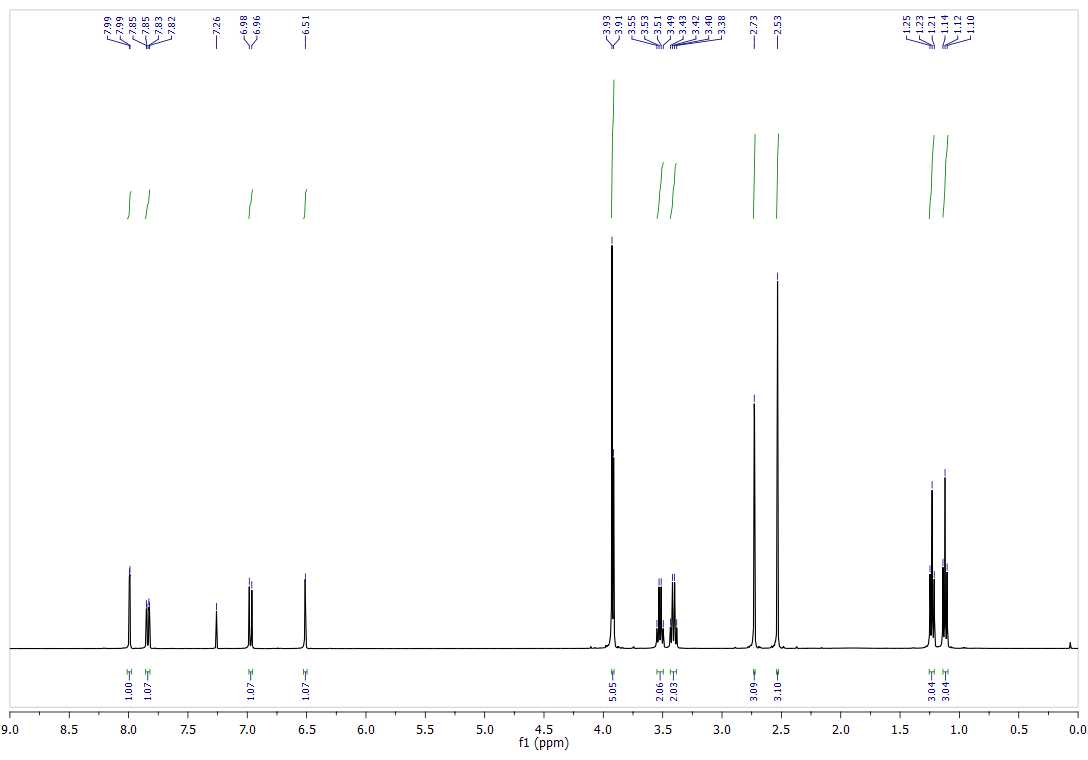


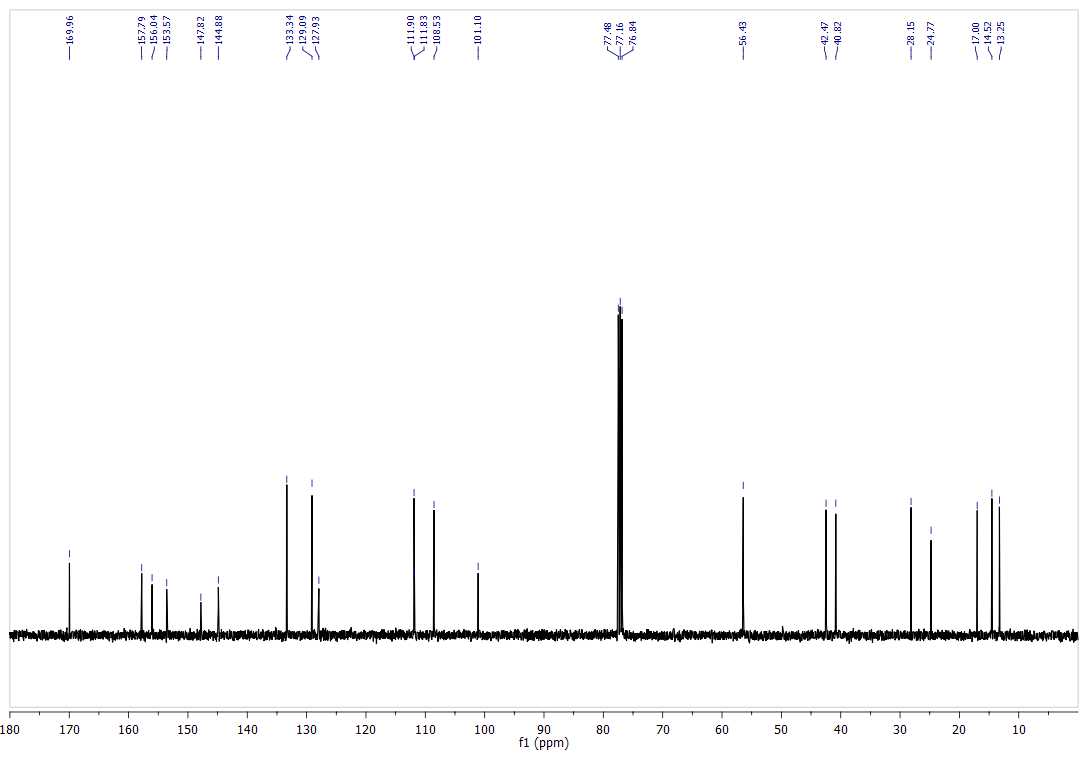


**G.** *N,N*‐Diethyl‐2‐{2‐[4‐methoxy‐3‐(tetramethyl‐1,3,2‐dioxaborolan‐2‐yl)phenyl]‐5,7‐dimethylpyrazolo[1,5‐*a*]pyrimidin‐3‐yl}acetamide


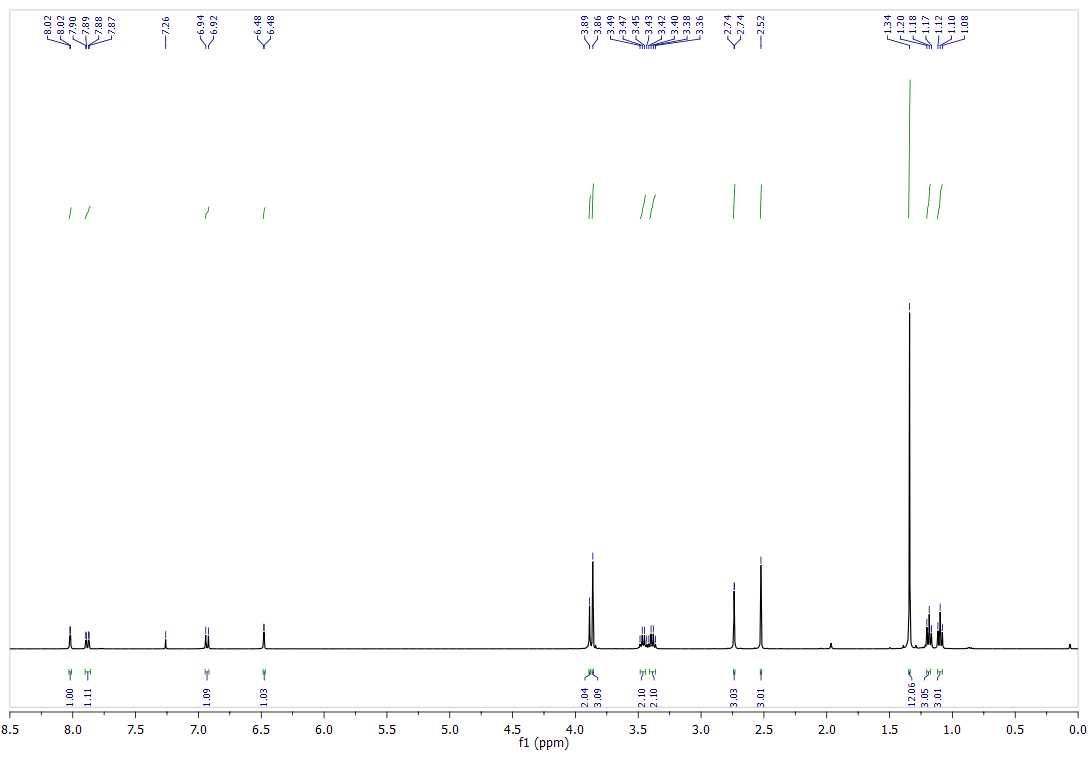


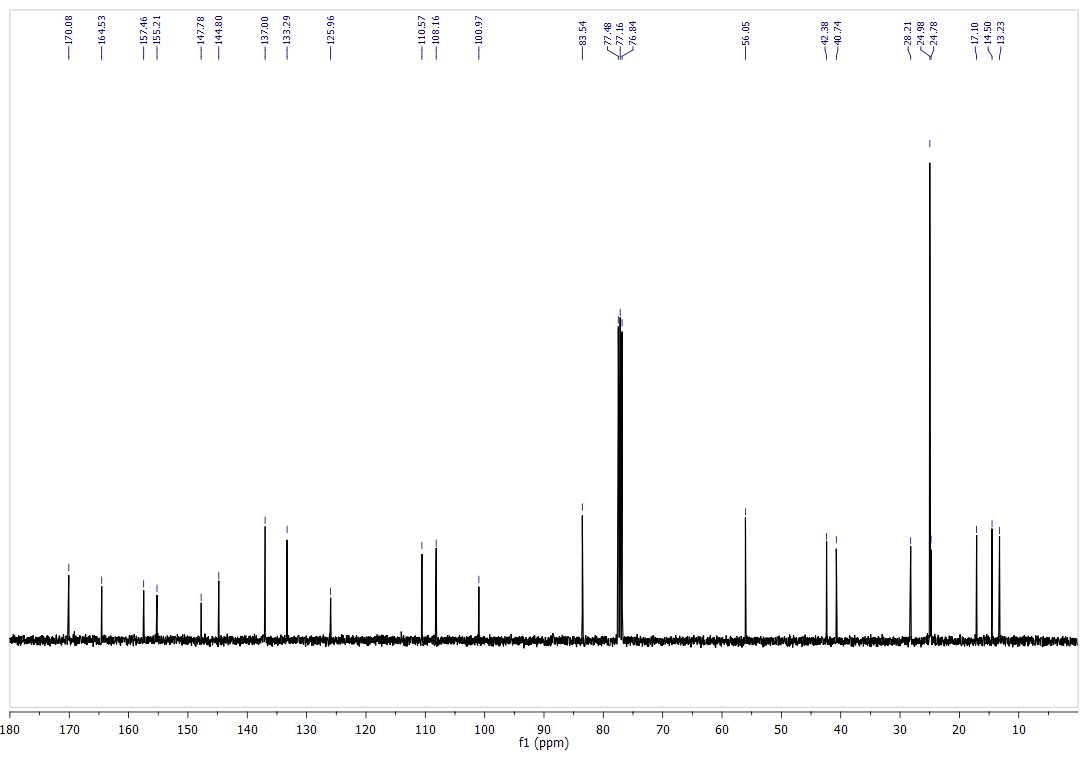


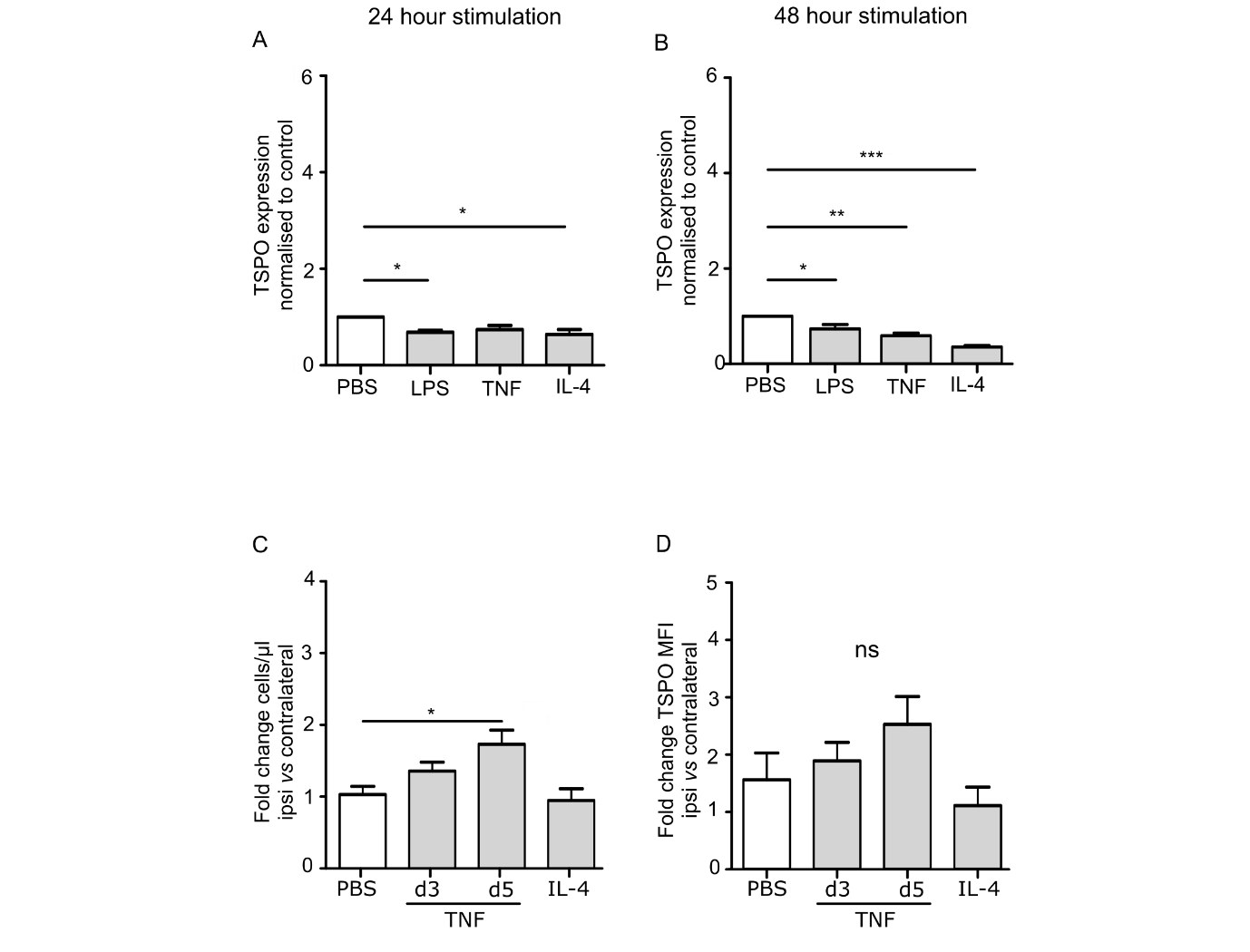


**Supplementary Figure 5.**

**Assessment of potential contribution of endothelial TSPO expression to TSPO imaging data**

TSPO expression in the endothelial cell line bEnd.3 (A, B) measured using flow cytometry following 24 or 48h stimulation with either LPS, TNF or IL-4. Graphs show TSPO expression normalized to control (n=3; ANOVA followed by Dunnett's multiple comparison test, *p<0.05, **p<0.005, ***p<0.001). Number of endothelial cells (C) expressing TSPO in ipsilateral hemisphere following injection of AdTNF or IL-4, normalized to contralateral. Median Fluorescence Intensity (MFI) of TSPO staining in endothelial cells (D) from ipsilateral hemisphere, normalised to contralateral side (n>6; ANOVA followed by Dunnett's multiple comparison test, *p<0.05, **p<0.005, ***p<0.001).


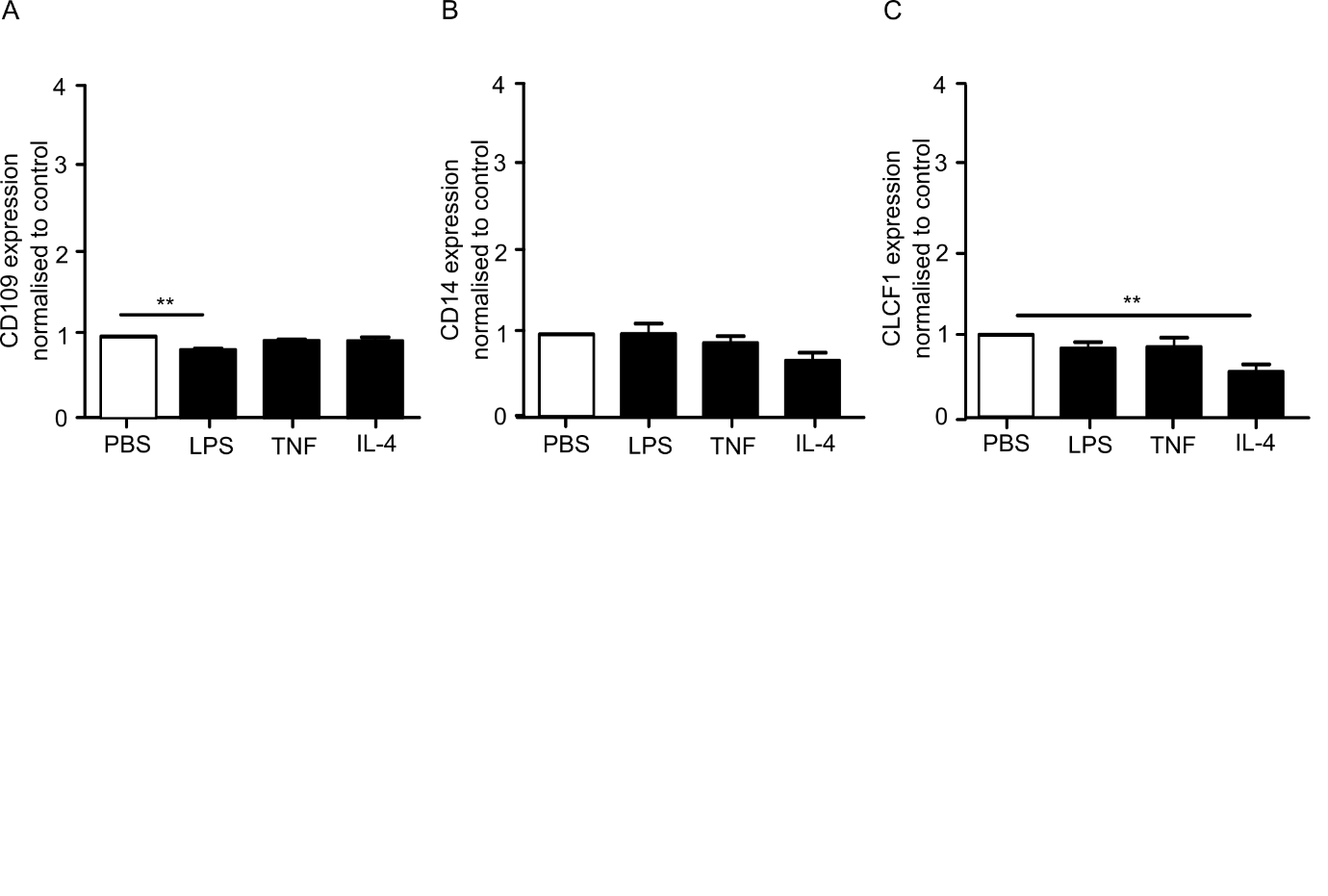


**Supplementary Figure 6.**

**CD109, CD14 and CLCF1 expression in astrocytes after pro- and anti-inflammatory stimulus**

CD109 (A), CD14 (B) and CLCF1 (C) expression in primary cultured murine astrocytes measured using flow cytometry following 24 hr stimulation with either LPS, TNF or IL-4. Graphs show CD109, CD14 and CLCF1 expression normalised to control (n=4; ANOVA followed by Dunnett's multiple comparison test, *p<0.05, **p<0.005, ***p<0.001).


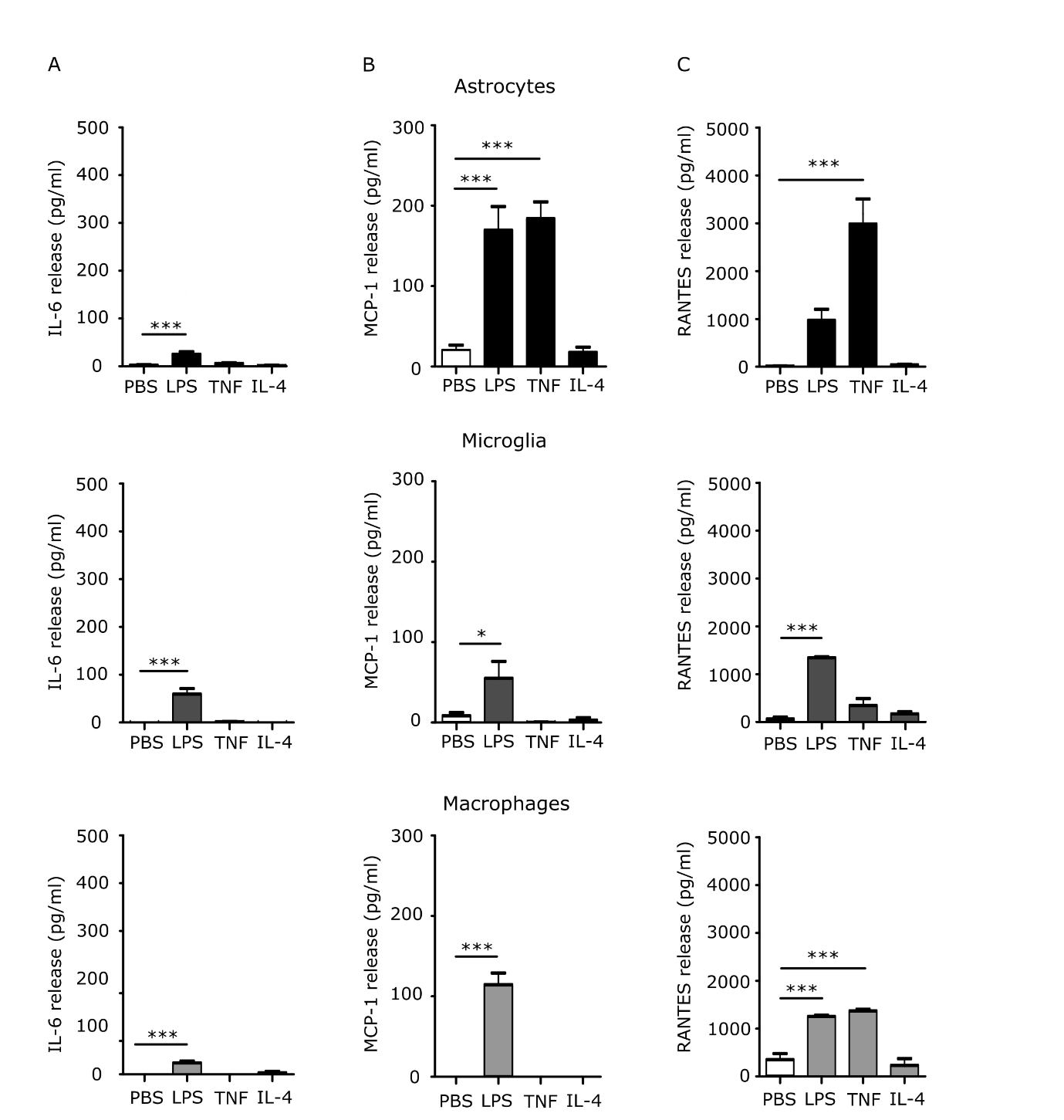


**Supplementary Figure 7.**

**Pro-inflammatory cytokine release from cultured astrocytes, microglia and macrophages 24h post-stimulation**

(A) IL-6, (B) MCP-1 and (C) RANTES release from primary cultured murine astrocytes (top row), microglia (middle row) and macrophages (bottom row) 24h after stimulation with LPS, TNF or IL-4 for 24h. Cytokine release into supernatant was measured 24h after stimulation, following washing of cells to remove residual stimulants (n>5, ANOVA followed by Dunnett's multiple comparison test, *p<0.05, **p<0.005 ***p<0.001)


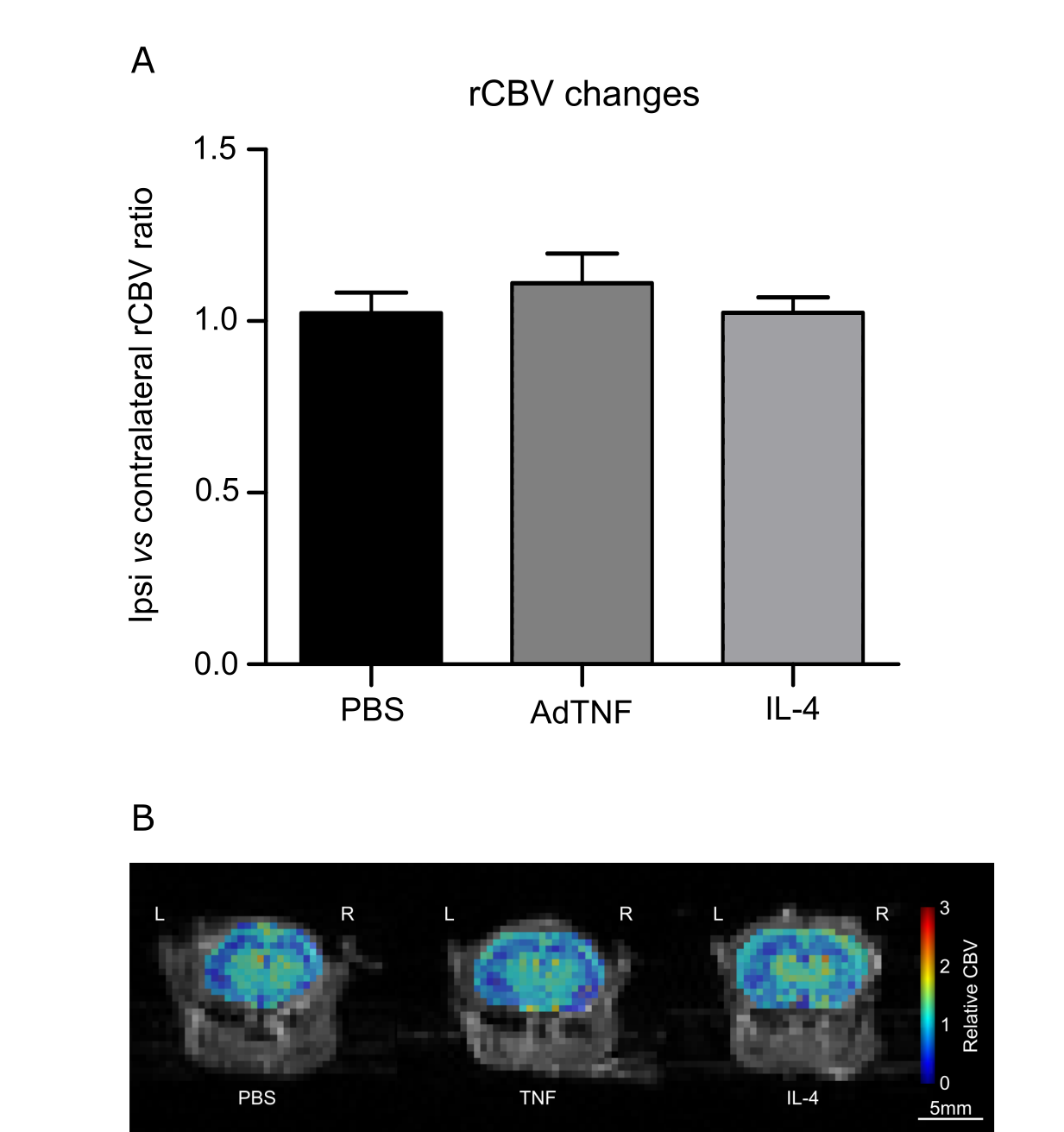


**Supplementary Figure 8.**

**Relative cerebral blood volume (rCBV) for injected striatum relative to control striatum**

(A) Graph of injected/contralateral CBV ratios in mice 5 days after AdTNF, or 24hr after IL-4 and PBS. N=3 per group. Value of 1.0 indicates no difference from contralateral control striatum. (B) Representative MRI images showing rCBV
